# Utility of Human Relevant Preclinical Animal Models in Navigating NAFLD to MAFLD Paradigm

**DOI:** 10.3390/ijms232314762

**Published:** 2022-11-25

**Authors:** Damien Chua, Zun Siong Low, Guo Xiang Cheam, Aik Seng Ng, Nguan Soon Tan

**Affiliations:** 1 Lee Kong Chian School of Medicine, Nanyang Technological University Singapore, 11 Mandalay Road, Singapore 308232, Singapore; 2 School of Biological Sciences, Nanyang Technological University Singapore, 60 Nanyang Drive, Singapore 637551, Singapore; 3 Radcliffe Department of Medicine, John Radcliffe Hospital, University of Oxford, Oxford OX3 9DU, UK

**Keywords:** nonalcoholic fatty liver disease (NAFLD), nonalcoholic steatohepatitis (NASH), metabolic dysregulation-associated fatty liver disease (MAFLD), hepatic fibrosis, hepatocellular carcinoma (HCC), cardiovascular disease (CVD), preclinical models, diet-induced obesogenic model, genetic models, chemically induced model

## Abstract

Fatty liver disease is an emerging contributor to disease burden worldwide. The past decades of work established the heterogeneous nature of non-alcoholic fatty liver disease (NAFLD) etiology and systemic contributions to the pathogenesis of the disease. This called for the proposal of a redefinition in 2020 to that of metabolic dysfunction-associated fatty liver disease (MAFLD) to better reflect the current understanding of the disease. To date, several clinical cohort studies comparing NAFLD and MAFLD hint at the relevancy of the new nomenclature in enriching for patients with more severe hepatic injury and extrahepatic comorbidities. However, the underlying systemic pathogenesis is still not fully understood. Preclinical animal models have been imperative in elucidating key biological mechanisms in various contexts, including intrahepatic disease progression, interorgan crosstalk and systemic dysregulation. Furthermore, they are integral in developing novel therapeutics against MAFLD. However, substantial contextual variabilities exist across different models due to the lack of standardization in several aspects. As such, it is crucial to understand the strengths and weaknesses of existing models to better align them to the human condition. In this review, we consolidate the implications arising from the change in nomenclature and summarize MAFLD pathogenesis. Subsequently, we provide an updated evaluation of existing MAFLD preclinical models in alignment with the new definitions and perspectives to improve their translational relevance.

## 1. Introduction

**Figure 1:**
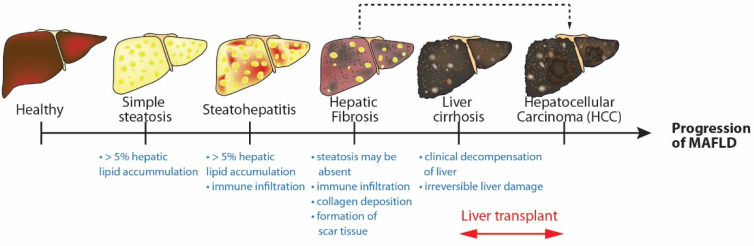
Progression of MAFLD (and NAFLD).

Nonalcoholic fatty liver disease (NAFLD) is an impending epidemic that affects a quarter of the world’s population [1]. It has rapidly become the leading cause that warrants liver transplantation in various parts of the world, with a prevalence of up to 40% in South–East Asian populations [2]. NAFLD is deeply connected with metabolic disorders, often regarded as the hepatic manifestation of metabolic syndrome, commonly seen in patients with obesity, hypertension, and diabetes [3]. NAFLD is an umbrella term that covers a spectrum of liver conditions that run from simple steatosis to severe conditions such as nonalcoholic steatohepatitis (NASH) and liver fibrosis. Left untreated, NAFLD patients run the risk of chronic liver injury and subsequent sequelae such as liver cirrhosis and hepatocellular carcinoma (Figure 1). In addition, an increased prevalence and incidence of chronic kidney disease (CKD) and cardiovascular disease (CVD) are also observed among these NAFLD patients, with the latter being the leading cause of death in NAFLD patients [4]. Despite the immense burden of disease, there is still no approved therapy for NAFLD; therefore, an in-depth understanding of the complex biology of NAFLD is warranted to advance rational therapeutic development.

As understanding of NAFLD expands with extensive research, older terminologies become increasingly inadequate to accurately describe the disease. Formerly, a two-hit hypothesis was used to describe the pathogenesis of NAFLD. Simple steatosis, characterized by hepatic lipid accumulation, was the first hit [5]. This notion was reinforced by the low proportions of patients who progress along the disease spectrum to develop NASH, coupled with experimental observations in mice that do not develop NASH spontaneously from solely dietary means. A secondary insult, such as lipopolysaccharide (LPS), was required for NASH progression in animal models, suggesting a need for a second hit that results in immune cell infiltration [6]. While this hypothesis holds a certain veracity, it is overly simplistic, considering the implications of metabolic aberrations and the involvement of multiple organs in NAFLD pathogenesis.

Consequently, the “multiple parallel hit” hypothesis was proposed in 2010 [7], explaining the tripartite interplay of adipose tissue, gut and liver in driving NAFLD inflammatory and fibrogenic responses. To date, many mechanistic and clinical studies have supported this hypothesis of NAFLD pathogenesis [8]. Expectedly, altered lipid metabolism is often observed in NAFLD patients, given that NAFLD is characterized by an abnormal accumulation of fat in hepatocytes. This phenomenon appears to be a defensive mechanism against a disturbed triacylglycerol (TAG) equilibrium [9]. Insulin resistance is a main determinant of such events, as TAG/free fatty acid (FFA) flux is controlled by the cellular insulin response, with the liver as a major insulin-responsive organ. As such, insulin resistance is a key contributor to the development of NAFLD, with a significantly increased likelihood of type 2 diabetes mellitus (T2DM) patients developing liver disease and with higher incidences of NASH progression [10,11]. Even in the absence of T2DM, patients with NASH saw decreased insulin sensitivity [12]. Consequently, cells are unable to cope with the increased lipid flux, leading to lipotoxicity and an overworked β-oxidation pathway. Consequently, mitochondrial dysfunction causes chronic oxidative and endoplasmic reticulum (ER) stress, commonly characterized by reactive oxygen species (ROS) accumulation [13].

Chronic lipid localization and ROS overproduction can activate inflammatory cascades, resulting in Kupffer cell and hepatic stellate cell (HSC) activation. These liver resident cells are responsible for hepatocyte necroinflammation and collagen deposition, respectively, driving further progression to NASH [14]. The immune system plays a critical role in NASH, as without adequate immune cell infiltration, a histological definition for NASH cannot be attained [15]. There is also a recurring theme: (a) cytokine involvement, such as IL-1β and TNF-α, both of which are proinflammatory cytokines evidenced to be key players in NASH development [16,17], and (b) metabolic memory displayed by the adaptive immune system [18]. The transition to NASH from simple steatosis is an important milestone of NAFLD development, which requires host immunological inputs, but the exact mechanism remains to be elucidated.

Blood from the gut accounts for a majority of the circulation going to the liver, allowing gut-derived factors to affect liver health [18]. Importantly, gut microbiota can contribute to this cross-organ interaction by letting toxins and metabolites slip past its cellular barriers [19]. Profound shifts in microbial interactions are known to negatively affect the host, resulting in dysbiosis, commonly seen in NAFLD patients [20]. Similarly, the lack of an “obesity-related” microbiome bestows immunity to obesity and NAFLD [21]. The plasticity of the gut microbiome has been documented and can be easily reshaped by diet or fecal matter transplant (FMT) [22], presenting it as a potential therapeutic target for the betterment of liver health.

Genetic variants have also been verified to affect the chances of developing NAFLD. Genome-wide association studies (GWAS) have identified several single nucleotide polymorphisms (SNPs) that are genetic drivers for NAFLD, namely, patatin-like phospholipase domain containing 3 (PNPLA3), transmembrane 6 superfamily member 2 (TM6SF2) and membrane bound O-acyltransferase domain-containing 7 gene (MBOAT7) [23]. Interestingly, these genes are strongly associated with FFA metabolism, encoding proteins involved in lipid metabolism processes, reinforcing the idea of perturbed metabolic parameters in the disease. However, genetic variations account for only a small fraction (<10%) of the patient population [24], suggesting a larger role for the environment and lifestyle in the pathogenesis of NAFLD.

However, the definitions of NAFLD presented several inadequacies when describing this liver condition. Firstly, it fails to reflect the critical role of a disordered metabolic system in the development of the disease as well as the involved metabolic risk factors and underlying comorbidities. In addition, the exclusionary framework of NAFLD (i.e., excluding excess alcoholic usage and secondary liver disease) does not fully capture the nuanced etiologies of the disease. To address this issue, experts have reconsidered clinical and research updates to the disease and proposed a new nomenclature: metabolic dysfunction-associated fatty liver disease (MAFLD) [25]. The new term acknowledges the principal state of systemic metabolic disarray and accounts for the possibility of other co-occurring chronic liver diseases, including alcohol use disorder. In view of the increasing prevalence of MAFLD, this move will reclassify patients with concomitant liver diseases, allowing consideration for metabolic issues to be resolved rather than ignoring them due to the exclusion criteria.

Collectively, insulin resistance, cell stress, ROS accumulation, inflammasomes, adipose tissue inflammation, gut microbiota dysbiosis, and gene–environment interactions reflect the dynamic and systemic interplay in MAFLD progression. Regardless, the core of MAFLD development remained centered around metabolic anomalies, such as dyslipidemia and insulin resistance, which is further emphasized in the renaming of the MAFLD nomenclature. In this review, we aim to summarize the key clinical implications of the NAFLD-to-MAFLD transition. Furthermore, with the changes in disease specifications, we review the utility of current preclinical animal models in recapitulating the disease.

## 2. MAFLD Definitions

**Figure 2:**
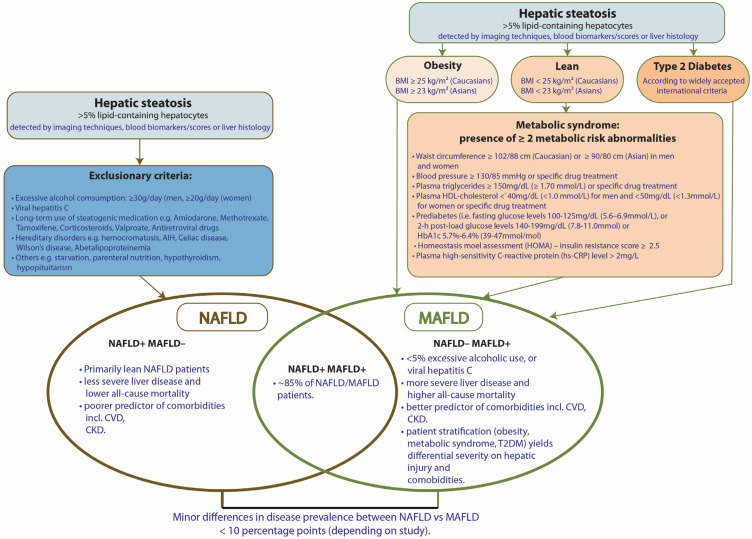
Definitions and clinical implications of the change in NAFLD nomenclature to MAFLD.

MAFLD diagnostic criteria are defined as the presence of hepatic steatosis, together with either obesity, the presence of T2DM or at least two metabolic abnormalities. Steatosis is the most defining feature of MAFLD and is histologically defined as having more than 5% lipid-containing hepatocytes [26] (Figure 2). At this stage, biopsy is usually not recommended, and hence, the use of ultrasound and magnetic resonance-based technology provides a suitable alternative for moderate to severely obese patients [27]. The use of obesity as an inclusion criterion stems from the link between obesity and clinically adverse outcomes [25], while T2DM, as mentioned previously, has historically a strong association with fatty liver progression [28]. Interestingly, the final criterion features a list of metabolic abnormalities that will identify metabolically unhealthy patients [29], who are at greater risk of liver injury and cardiovascular risk than obese yet healthy individuals [30]. This acts as a flexible wildcard that provides a safety net for capturing NAFLD subtypes that were once difficult to stratify, such as lean NAFLD, where nonobese patients who are metabolically unhealthy are found to have liver steatosis [31,32] (Figure 2).

Another revolutionary change proposed by the panel was to view MAFLD as a single overarching term without stratification into NASH and non-NASH progression. Historically, the progression to NASH marks an important milestone in disease progression and a worsening of patient prognosis [26]. Recently, longitudinal studies have associated an increase in the grade of activity with fibrosis progression, while fibrosis regression showed a similar decrease in the activity grade despite continued steatohepatitis. This highlights the importance of the grade of activity in predicting patient prognosis rather than the focus on the dichotomous classification of the presence of liver inflammation. Viewing MAFLD as a single progression continuum wholly removes the limitations of the histological scoring system not being able to delineate NASH [33], allowing severity to be viewed quantitatively via grade of activity and stage of fibrosis. At the same time, this move is in concordance with the abolishment of an oversimplistic “two-hit hypothesis”, building momentum toward a new understanding of the disease with clearer clinical data at hand.

The new nomenclature seeks to better reflect the etiology of fatty liver disease, which is largely metabolic driven. These involve several inclusion criteria, a stark change from a previously “negative” diagnosis. Importantly, the new basis for diagnosis acknowledges that patients with concomitant liver disease allow greater flexibility in encompassing the heterogeneity of fatty liver subtypes and a single overarching term for disease progression focusing on disease severity described by grade of activity and stage of fibrosis.

## 3. Clinical Landscape with New MAFLD Criteria

The obvious difference in nomenclature often mentioned involves the removal of exclusionary criteria on the lack of alcohol use and other associated diseases, such as viral hepatitis. Consequently, the MAFLD cohort will then expand into this range of patients. Furthermore, a more metabolic disorder-centric criterion is emphasized above other etiologies. Herein, the question lies in how this would change the clinical landscape of disease.

Generally, the global prevalence of MAFLD and NAFLD is reported to vary from 30–40%, based on different studies, with the difference in diagnosis under the respective MAFLD and NAFLD criteria being <10 percentage points across several cross-sectional studies. Consistently, these studies reported that patients diagnosed with both MAFLD and NAFLD (overlapping cohort) account for a large majority (80–90%) of all NAFLD and MAFLD diagnosed patients [34,35,36,37]. Superficially, this indicates that the change in nomenclature only redefined a minority of the patients. Despite these minor differences at the macroscopic level, the MAFLD criterion is associated with higher liver injury biomarkers (i.e., AST, ALT), further progression of disease (i.e., fibrosis) [38,39,40] and higher cardiometabolic risk.

Within the MAFLD group, it was found that MAFLD with excessive alcohol consumption or hepatitis B infection accounted for only <5% of the entire cohort [34], indicating that the increased comorbidity risk factors are not entirely due to the previously excluded patients (in NAFLD) but are also due to a higher emphasis on underlying metabolic dysregulation [41,42]. Interestingly, further stratification of MAFLD into its diagnostic criteria level (i.e., MAFLD with obesity or diabetes or metabolic syndrome) indicated different severities of all-cause mortality [43]. The same approach found increased hepatic disease progression, especially in the MAFLD with diabetes group [44]. This suggests that future studies should include multilevel interrogation of clinical data to provide more informed prognostication of patients, underscoring the heterogeneity of the disease even after redefinitions.

Progression of disease into fibrotic liver stages represents a key clinical and therapeutic turning point as the strongest predictor of liver-associated mortality and indicator of worsening of disease [45]. In a recent 2021 Rotterdam study investigating changes in nomenclature for liver stiffness, it was found that MAFLD-only patients were associated with a higher fibrosis prevalence [46]. Studies in Japan [47], Korea [44], China [48], the United States [36,39,49] and Sri Lanka [49] also found the same associations. This effect was especially found in MAFLD patients who are nonobese [50,51]. Conversely, NAFLD-only patients who presented only simple steatosis and no metabolic dysregulation were found to be at lower risk of developing advanced disease [52]. In such cross-sectional studies, there were a few studies that showed little to no difference in the prevalence of increased fibrosis risk in all MAFLD+ NAFLD+ (overlapping diagnosis) patients. Particularly, the lack of diagnostic data recorded for the 3 categories of MAFLD resulting in the exclusion of many MAFLD+ patients has been highlighted [53]. This is an important gap that future MAFLD clinical studies need to fill to obtain a clearer picture of the MAFLD-NAFLD landscape. Nonetheless, the majority of the studies indicated promising use of MAFLD diagnoses to capture patients with a higher risk of disease severity.

In NAFLD, it has been well established that increased severity in liver disease progression is highly correlated with increased cardiometabolic risk [54]. Cardiovascular disease (CVD) has been heralded as the main cause of death in patients with NAFLD (approximately 45% of deaths) [55,56]. CVD involves various cardiomyopathies that are usually fatal. These include left ventricular contractile dysfunction and hypertrophy, atherosclerotic CVD, cardiac arrhythmias, and ischemic stroke [57]. In a seminal Korean nation-wide cohort study involving 9.5 million participants, it was found that the MAFLD-only group significantly better predicts cardiovascular events [58,59]. A global systematic review of 12 million individuals [60] and a study in Xinjiang, China, also revealed a similar trend [61]. While Guerreiro et al. identified that the increased MAFLD-associated cardiovascular risk is attributed to concomitant hepatitis viral infection etiology [62], Matsubayashi et al. found that MAFLD with T2DM showed greater coronary artery disease risk [63]. These results were consistent with Tsutsumi et al., where the employment of the generalized estimating equation (GEE) approach in their cohort study reported a high risk of MAFLD-associated CVD risk compared to that of NAFLD, and these were attributed to metabolic dysfunction rather than alcohol consumption etiologies [64]. Taken together, these results suggest that the MAFLD criterion is predictive of CVD risk, primarily due to metabolic factors in the disease, rather than other etiologies, such as alcohol use.

In addition to CVD, chronic kidney disease (CKD) is another commonly reported extrahepatic manifestation of MAFLD. The severity of liver fibrosis has been found to promote the prevalence and incidence of CKD in NAFLD patients in several previous studies [65,66,67]. Mainly, a low estimated glomerular filtration rate of <60 mL/min/1.73 m^2^ and the presence of proteinuria are used to diagnose CKD in patients [68]. Consistently, the MAFLD criterion better predicted patients with a higher prevalence of CKD [69]. However, no studies performed disease stratification to interrogate the contribution of metabolic-associated etiologies or viral hepatitis and alcohol consumption to the increased MAFLD-associated CKD prevalence, all of which are independent risk factors for CKD [70]. Hence, further cohort studies are definitely required to investigate the relationship between MAFLD and CKD.

Overall, although more studies are needed, most studies to date suggest better enrichment of patients with a higher risk of (i) liver disease progression and mortality, (ii) CVD, and (iii) CKD. Notably, various studies highlighted the potential value-add in stratifying patients into various etiologies of the MAFLD definition, i.e., metabolic syndrome, obesity or T2DM, or further permutations among these groups. Primarily, the differential outcomes for these various risks could provide important information in prognostication, management, and care for patients in the future. To this end, these indications further accentuate the complexity and systemic nature of the disease. Accordingly, it is important to understand how current animal models adhere to these stratifications. Proper investigation of animal models in respective categories allows for the shift toward demystifying the biological basis of these differential disease outcomes and implications. Further mechanistic studies into this disease require strong emphasis on systemic processes and interorgan metabolo-interactomes as well, rather than independent pathologies such as simply fibrotic liver. In Section 5, we will discuss how current models fit into MAFLD definitions for closer animal model translatability.

## 4. Pathogenesis of MAFLD

Currently, most studies on MAFLD pertain to its clinical implications for the change in nomenclature. While the proposed changes are still in their infancy, novel differential pathogenesis pathways of MAFLD and NAFLD have not been reported. Hence, the mechanisms underlying the disease progression of MAFLD are currently understood to be largely similar to those of NAFLD in terms of systemic metabolic dysregulation. Despite this, recent research has shed light on the biological relevance of using MAFLD nomenclature in describing pathogenesis. For instance, in NAFLD patients, elevated serum ethanol levels were found to be significantly higher than those in subjects without NAFLD. This is associated with changes in gut microbiome-associated alcohol dehydrogenase activity associated with enrichment of Lactobaccillaceae taxa in the gut [71]. Similarly, NAFLD and MAFLD share many similar metabolic and inflammatory derangements [72]. With such evidence arising, it will be sensible to rethink MAFLD despite its etiological heterogeneity. Several nuanced mechanisms present in MAFLD will be highly important to assess the translatability of MAFLD preclinical models.

As such, this section aims to summarize important pathogenesis pathways relevant to MAFLD. Given that previous studies were based on NAFLD nomenclature, these studies will be mentioned using NAFLD in place of MAFLD to adhere to the stringency of definitions.

### 4.1. Ectopic Lipid Spillover Impairs Insulin Sensitivity and Generates Lipotoxicity in the Liver

Obesity and diabetes are well-known risk factors in MAFLD progression, and reduced insulin sensitivity coupled with perturbed lipid metabolism drives lipotoxic metabolic stress [73]. Traditionally, body mass index (BMI) is a widely used metric for obesity that measures total body fat (consisting of approximately 80% subcutaneous and 29% visceral fat). An obese BMI is considered ≥30 for Caucasians and ≥25 for Asians in the clinical setting [74]. Nonetheless, experts are divided on BMI as an index for obesity, as it insufficiently captures the cardiometabolic risk due to increased adiposity [75]. Waist circumference (WC), which measures abdominal adiposity, better predicts increased visceral adiposity and is positively correlated with fatty liver [76] and hepatic insulin resistance [77]. On the flip side, being obese by the BMI standard does not always imply higher cardiometabolic risk. This is corroborated by a large-scale cohort study involving more than 9000 individuals who found that those with NAFLD who were lean by BMI but obese by WC had a higher risk of all-cause and cardiovascular mortalities [78]. The longstanding perception that obesity by BMI index is the primary cause of fatty liver often underestimates the propensity of another subset of lean/nonobese but metabolically unhealthy individuals to develop MAFLD [79]. Therefore, to better encompass both the obese and nonobese subpopulations, there is a need to disparate the total fat distribution, more precisely the visceral adipose tissue (VAT), given its disproportionately significant contribution to insulin resistance and metabolic syndrome than subcutaneous fat [80,81].

**Figure 3:**
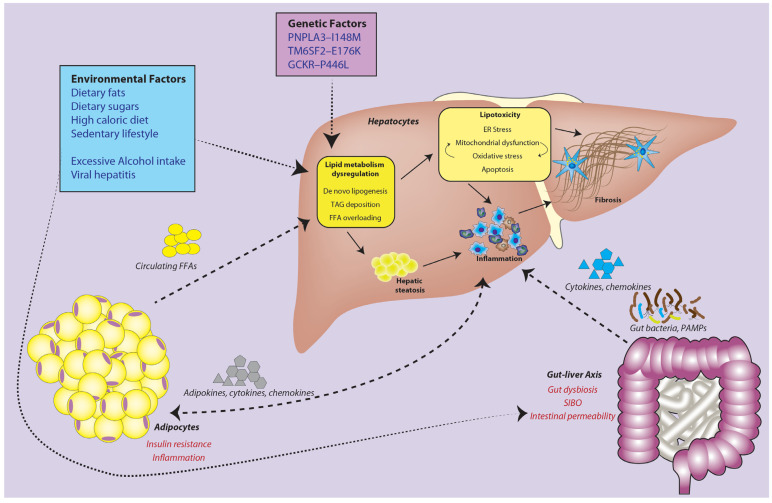
Summary schematic of MAFLD pathogenesis.

Many factors can instigate excessive lipid deposition; prolonged nutrient overloading [82], a sedentary lifestyle, and elevated de novo lipogenesis [83] promote increased deposition to both subcutaneous adipose tissue and VAT [84]. Various genetic and epigenetic players could limit the number and expansion capacity of adipocytes [85,86,87,88]. In particular, when primary storage capacities in SAT have been overloaded, ectopic lipid spillover to VAT and non-adipose organs (e.g., liver, skeletal muscles and pancreas) occurs. This results in deleterious metabolic and cardiovascular risks by promoting inflammation, inhibiting insulin signaling [89], and impairing glucose metabolism, culminating in insulin resistance [90]. Excess FFA in VAT is channeled into the liver via the portal circulation, resulting in hepatic fat deposition or liver steatosis [81], underpinning lipid metabolism dysregulation and lipotoxicity. Past studies generally characterized steatosis as excessive triacylglyceride (TAG) deposition in the cytoplasm of hepatocytes (Figure 3). Under normal physiological conditions, TAG assembly precedes the secretion of TAG-enriched very-low-density lipoprotein (VLDL-TAG), which occurs under fasting conditions [91], thus serving as a protective function against lipotoxicity from fatty acids [92,93]. Therefore, TAG accumulation reflects a state of hepatocyte FFA overloading, whereby a subset of the lipid species, such as saturated FFA, i.e., palmitate (C16:0) from dietary sources, lysophosphatidylcholine (partially hydrolyzed phosphatidylcholine), ceramide (de novo synthesis from palmitoyl co-A), free cholesterol (enhanced synthesis) and bile acids (degradation of cholesterol), are lipotoxic and activate several signaling pathways of apoptosis, leading to liver damage and steatohepatitis [94,95].

### 4.2. MAFLD Risk Amplification by Genetic Predisposition and Adiposity

The advent of genome-wide association studies (GWAS) has led to the identification of polymorphisms in several genes involved in regulating hepatic lipid metabolism that are strongly associated with increased susceptibility to NAFLD and NASH. PNPLA-3 is expressed in the liver and is implicated in the remodeling of phospholipids of lipid droplets. PNPLA-3 is a lipogenic transacetylase that hydrolyses TAG and catalyzes the transfer of polyunsaturated fatty acids from di- and tri-acylglycerols to phosphocholine [96,97]. The PNPLA-3 variant rs738409 (C > G), which encodes PNPLA3 I148M (substitution of isoleucine to methionine at position 148), resists degradation and is associated with increased hepatic fat content, with a high frequency of this risk allele in concordance with the high prevalence of NAFLD among Hispanics [98]. Mechanistically, impaired clearance of this ubiquitination-resistant form of PNPLA3 promotes hepatic steatosis by accumulating on hepatic lipid droplets. The ablation of PNPLA3 I148M by either shRNA knockout or PROTAC-mediated proteasomal degradation can reverse steatosis [99]. Transmembrane 6 superfamily member 2 (TM6SF2) is present in the endoplasmic reticulum (ER) and the Golgi complex of hepatocytes and is involved in the multistep process of VLDL secretion. It facilitates the transfer of neutral lipids from lipid droplets into VLDL particles within the ER lumen, en route to, and within the Golgi complex [100]. Individuals with the TM6SF2 variant rs58542926 (C > T), which encodes an inactivated form of TM6SF2 E176K, have elevated liver TAG and decreased serum lipoprotein apoB levels [97]. The synergy between obesity and genetic variants may have a role in influencing NAFLD susceptibility and progression, whereby adiposity can amplify the effects of risk alleles by increasing their expression. At the same time, the energy surplus serves to fuel hepatic steatosis [101]. Another implicated enzyme is glucokinase regulator (GCKR), which is involved in de novo lipogenesis. The missense mutation GCKR P446L, rs1260326, results in the elevation of malonyl Co-A, which is a substrate for hepatic lipogenesis [97]. To this end, accumulating evidence suggests that genetic depositions and obesity are interrelated in a feedback loop influencing MAFLD susceptibility, where genetic mutations can drive obesity and vice versa [101].

The concept of “missing heritability” in complex diseases, including NAFLD, has been a long-standing problem since the advent of GWAS. *Heritability* estimates the robustness of genes in predicting traits. Regarding NAFLD, the collective variants of over 100 loci uncovered by candidate-gene association studies merely explain a small fraction of the phenotypic variance of NAFLD and NASH [102], implying that the heritability of gene polymorphisms is relatively modest. While the topic is beyond our scope, a review by Sookoian and Pirola summarizes factors such as structural variations, mitochondrial genetics, epigenetic inheritance, and the microbiome in the missing heritability of NAFLD and NASH [103].

### 4.3. Steatosis Causes Endoplasmic Reticulum and Mitochondrial Dysfunction in Hepatocytes

Endoplasmic reticulum (ER) stress and oxidative stress play a significant role in the pathogenesis of MAFLD. The ER is a major site of lipid synthesis and VLDL assembly in hepatocytes, where different ER-localized enzymes orchestrate the processes. Sterol regulatory element-binding proteins (SREBPs) are ER-membrane localized transcription factors containing a cytosolic N-terminal domain that activate gene transcription when it binds to sterol response elements (SREs) and regulates fatty acid and cholesterol synthesis. Usually, SREBPs are held inactive by SREBP cleavage-activating protein (SCAP) and ER-resident protein known as insigs (insulin-induced gene). When sterol is low, insigs dissociate and allow the SREBP-SCAP complex to migrate to the Golgi, where SREBPs are processed into their transcriptionally active forms [104]. Fructose [105], C16 ceramide [106] and oxidative stress [107] are known to perturb ER proteostasis, resulting in the accumulation of unfolded/misfolded proteins within the ER. This triggers the activation of the unfolded protein response, facilitated by three different pathways: protein kinase RNA-like-endoplasmic reticulum kinase-eukaryotic initiation factor 2 alpha (PERK-eIFα), inositol-requiring enzyme 1 alpha/X-box binding protein 1 (IRE1α/XBP1), and activating transcription factor 6 alpha (ATF6α), which collectively orchestrates the upregulation of protein-folding capacity and reduces global protein synthesis [108]. As such, more SREBPs become active, and consequently, lipogenesis is elevated. Moreover, lipid accumulation initiates a positive feedback loop, leading to chronic ER stress.

Mitochondria are another crucial organelle in hepatocytes directly affected by steatosis given their central role in the beta-oxidation of fatty acids. Increased flux of fatty acids increases the pool of electron carriers such as NADH and FADH_2_ due to an enhanced rate of fatty acid oxidation and tricarboxylic acid cycle [109]. Overbearing of the electron transport chain (ETC) manifests into a state of oxidative stress where the leakage of electrons is beyond the normal capacity of electron scavengers, leading to the buildup of damaging levels of reactive oxygen species (ROS), such as hydroxyl radicals and superoxide anions, that can react with intracellular biomolecules [110]. Clinically, hepatic mitochondria are remodeled in NAFLD [111], with increased ROS production, decreased antioxidant capacity, increased fragmentation and calcium overload [112]. Furthermore, oxidative stress can exacerbate mitochondrial dysfunction in a vicious cycle, leading to more lipid accumulation and ER stress.

### 4.4. Unresolved Stresses Can Trigger Apoptosis, Contributing to Inflammation and Fibrosis

Sustained insults from lipotoxicity-inducing agents, fueled by steatosis, insulin resistance, and a saturated high-fat and high-fructose diet, cause a long-drawn ER stress-mediated UPR beyond its beneficiary threshold, destining the damaged hepatocytes for cell death [113]. Apart from downregulating protein synthesis and increasing the protein-folding capacity, the UPR proteins IREα and PERK-elF2α also activate C/EBP homologous protein (CHOP), a proapoptotic transcription factor. CHOP functions by repressing antiapoptotic proteins such as BCL2, BCL-XL, and MCL-1 while upregulating proapoptotic proteins such as BAK and BAX, causing the release of the apoptotic factor cytochrome C via mitochondrial permeabilization. In addition, it also upregulates death receptors 4 and 5 (DR4, 5) to sensitize the cell to extrinsic cell death stimuli, concomitantly causing a caspase cascade that eventually leads to cell death [114]. However, conflicting experimental observations on ER stress and CHOP involvement in NAFLD progression based on ER stress suppression [115,116] and CHOP ablation effects [117,118] have yet to be resolved. Nevertheless, this reflects the multifactorial nature and heterogeneity of the disease pathophysiology, making therapeutic efforts challenging.

Hepatocellular death is a histological hallmark of an advanced stage of NAFLD and sets the stage for inflammation and fibrogenesis, representing NASH. Clinically, the products of the intrinsic apoptosis pathway, such as the caspase cleaved intermediate filament cytokeratin 18 (CK18) [119], and the extrinsic pathway, such as soluble Fas and its sFas and sFasL [120], serve as noninvasive apoptosis biomarkers for the onset of NASH. In particular, damaged CK18 can form cytoplasmic inclusions known as Mallory-Denk bodies (MDBs) in ballooned hepatocytes, mediated by stress protein p62/Sequestosome-1 [121]. Ballooned hepatocytes can be determined with histological techniques such as oil red O positivity (presence of fat droplet accumulation), hematoxylin and eosin (H&E) staining (cellular enlargement with a rarefied cytoplasm) and anti-K18 immunostaining (diminished K18 expression with stained MDBs) [122]. The balloon degeneration of hepatocytes accompanied by the downregulation of heat shock protein 27 (HSP27) is speculated to demonstrate an impaired ability of hepatocytes to cope with metabolic stressors [123], indicating a worse prognosis.

Hepatocellular death, lipotoxicity, and gut bacteria-derived endotoxins can stimulate Kupffer cells to initiate inflammation, recruit immune cells such as monocyte-derived macrophages and neutrophils, and promote tissue remodeling in the liver. Apoptotic cells express danger-associated molecular patterns on their surface and release extracellular vesicles containing CXCL10 and TRAIL [124], which are sensed by Kupffer cells through pattern recognition receptors and internalization of vesicles, respectively. This triggers an M1-type, proinflammatory polarization of macrophages that engages in cell debris clearance by phagocytosis and the release of proinflammatory cytokines and chemokines such as CCL2 to recruit monocyte-derived macrophages and neutrophils to assist in the resolution of injury and promote liver regeneration [125]. Nonetheless, elevated macrophage and neutrophil counts are also correlated with liver injury severity [126,127,128], whereby the destructive effects of inflammation outweigh the beneficial effects in this case.

The presence of fibrosis—which is the accumulation of extracellular matrix (ECM) proteins such as collagen, fibronectin, laminin—is a distinguishing hallmark of NASH resulting from an aberrant wound-healing response from persistent injury [129]. Here, hepatic stellate cells (HSCs) play a significant role in fibrogenesis. Under normal physiological conditions, HSCs residing in the perisinusoidal space (space of Disse) display a quiescent cell state and function as a vitamin A storage site. Upon activation, HSCs transdifferentiate into myofibroblast-like cells and accumulate at damage sites to synthesize and deposit ECM materials. This conversion is stimulated by transforming growth factor-β (TGF-β), which is released from Kupffer cells, monocyte-derived macrophages, and activated HSCs themselves, in an autocrine/paracrine fashion to amplify ECM deposition [130].

### 4.5. Gut Dysbiosis Is Implicated in Disrupted Energy Homeostasis and Hepatic Inflammation

Several lines of evidence from various gut microbiota studies have demonstrated its contributory role in the onset and progression of NAFLD; the grafting of fecal content from donor mice that are highly responsive to a high-fat diet into germ-free mice resulted in experimentally induced NAFLD [131], with gut modulation by antibiotic administration [132] and synbiotic supplementation [133] correlated with lower lipogenesis and diminished hepatic steatosis. Patients with NAFLD and NASH display an altered intestinal microbiome characterized by small intestinal bacterial overgrowth (SIBO) [134,135], lower bacterial diversity [136], enrichment of Enterobacter and Streptococcus [137], and reduced Bacteroidetes [138]. A sedentary lifestyle, lack of physical activity, Westernised diet, and antibiotic usage are strong attributes to the perturbation of the gut microbiota. Given the promising prospect of gut modulation in ameliorating the progression and even possibly preventing the onset of NAFLD, more research in this area is still warranted in elucidating the mechanism of the gut microbiota and characterizing the gut microbial composition of NAFLD, which can aid in the development of risk assessments and therapeutic strategies.

An elevated ratio of *Firmicutes* to *Bacteroidetes* is responsible for the disruption of energy homeostasis involving triacylglycerol metabolism and fatty acid oxidation, which are implicated in steatosis. Angiopoietin-like 4 (ANGPTL4), previously known as fasting-inducible adipose factor, is expressed in adipose tissue, liver, muscle and intestine and is an essential regulator of triglyceride metabolism, carrying out this role by inhibiting the enzymes lipoprotein lipase and pancreatic lipase during fasting [139]. Loss of ANGPTL4 can result in increased total body and adipose tissue mass and visceral adipose tissue inflammation and thus has been implicated in obesity [140]. Notably, the “obese” microbiota can suppress the expression of ANGPTL4 mRNA in the fecal transplant recipient, enhancing lipogenesis and TAG levels in the liver [141]. However, the underlying mechanism remains to be identified. Adenosine monophosphate kinase (AMPK), which is expressed in the liver and skeletal muscles, is known as a metabolic master switch that can phosphorylate acetyl-CoA carboxylases (ACC-1, -2) to regulate fatty acid oxidation in response to cellular energy changes [142]. Abolishing AMPK expression can result in insulin resistance and hepatic steatosis [143], making it a suitable therapeutic target to treat type II diabetes and obesity [144]. While the role of high-fat diet-induced gut dysbiosis in AMPK inhibition in the liver has been validated by various gut modulation strategies [145,146,147] that reversed this suppression, the underlying mechanism remains ambiguous. The translocation of microbial endotoxins such as LPS that are involved in AMPK inhibition-mediated gut barrier dysfunction [148] via the gut-liver axis could influence AMPK activity in the liver.

A compromised gut barrier can increase the translocation of gut bacteria and endotoxins via the gut-liver axis and trigger hepatic inflammation. Typically, the translocation of gut bacteria and pathogen-associated molecular patterns (PAMPs), such as LPS, is effectively prevented by the tough defenses of the mucosal lining of the gut and tightly packed enterocytes reinforced by tight junction proteins, such as occludin and ZO-1. Secretory cells found at the bottom position of small intestinal crypts known as Paneth cells release different antimicrobial peptides, such as α-defensins, to limit pathogen colonization and bacterial-epithelial cell contact [149]. A high-fat diet can compromise the protective mucous layer of the intestinal lining, stimulate the proinflammatory signaling cascade that promotes barrier-disrupting cytokines and allow the penetration of pathogens across the thinned mucous layer, where they can alter tight junctions and translocate across the gut [150]. In a similar fashion, chronic fructose intake is associated with SIBO, which is causal in poorer gut integrity [151]. Mice with induced colitis fed a high-fat diet fared worse than those fed a high-fat diet alone, displaying more severe hepatic steatosis and steatohepatitis, further demonstrating the link between a compromised gut defense and NAFLD progression. A “leaky gut” permits the continuous flux of bacterial components into the liver beyond the threshold clearance. Mechanistically, these leaked bacterial components are recognized as PAMPs by different toll-like receptors (TLRs) on Kupffer cells [152]. LPS recognition by TLR4 triggers a signaling cascade that leads to the activation of the nuclear factor-kappa B (NF-κB) pathway, which can upregulate the production of tumor necrosis factor α (TNF-α), contributing to a chronic inflammatory response.

Comparative analysis of the gut microbiota composition of healthy, obese and NASH cohorts revealed a distinct difference: the marked increase in the abundance of *Escherichia*, an ethanol producer, could help to explain why NASH patients often have elevated blood ethanol levels [153]. The implicit role of endogenous alcohol in NASH manifestation raises the question of whether it is still appropriate to be termed “nonalcoholic”.

### 4.6. Recognizing the Role of Excessive Alcohol Intake in Contributing to MAFLD Progression

The rebranding of NAFLD to MAFLD reinforces the contribution of excessive alcohol intake to the disease etiology. One of the primary effects of chronic heavy alcohol consumption (>60 g of pure alcohol on one occasion [154] for more than five years) or binge drinking (>40 g for women and >50 g for men within two hours) is the elevated risk of obesity attributed to its high caloric content (generating 7.1 kcal per gram of ethanol), leading to a higher BMI and WC, and is found to have a stronger link in men at different ages than in women [155]. The pathophysiology of alcohol-induced liver diseases closely resembles that of NAFLD, despite the term nonalcoholic implying a distinct etiology [156]. Ethanol metabolism occurring in the liver alcohol can influence lipid metabolism—suppression of fatty acid β-oxidation, upregulation of fatty acid transporters, aberrant TAG synthesis by upregulation of lipin-1, contributing to hepatic steatosis [157]. Alcohol use is also implicated in the disrupted intestinal barrier, resulting in increased flux of bacterial endotoxins into the liver to stimulate the Kupffer cell, resulting in hepatitis. Alcohol intoxication in individuals with fatty liver disease can rapidly induce a change in the profile of circulating lipids, suppressing TAG clearance and increasing hepatic lysophosphatidylcholine, which are drivers of ER and oxidative stress, leading to the apoptosis of hepatocytes [158].

### 4.7. Chronic Viral Hepatitis Is Implicated in Impaired Lipid Metabolism in Hepatocytes

Chronic hepatitis C (CHC) is another widespread liver disease that affects approximately 3% of the global population, with a high seroprevalence in Asian and African countries, and is also one of the leading risk factors for cirrhosis and liver cancer [159]. The major transmission routes of hepatitis C virus (HCV) include blood transfusion, intravenous and intranasal drug usage, and unsafe therapeutic injections [160]. The vast majority of acute HCV infections are asymptomatic and are primarily detected through screening programs among the high-risk groups for HCV seropositivity, and usually, approximately 80% of those infections become chronic [161]. The persistence of HCV mRNA and viral proteins in chronically infected individuals can modulate transcription programmes related to lipid and glucose metabolism, such as increased de novo lipogenesis, downregulated lipoprotein exportation and impaired β-oxidation [162], which can induce the development of hepatic steatosis—a common feature in many CHC patients [162,163].

### 4.8. Deficiency in Choline Results in Mitochondrial Dysfunction in MAFLD

Choline is an essential macronutrient found in food from plant and animal sources and is involved in neurotransmitter synthesis, cell membrane signaling, lipid transport and methyl-group metabolism [164]. Choline deficiency in the development of NAFLD is well established by the methionine/choline-deficient (MCD) diet and is a classical dietary model of NASH [165], whereby mitochondrial dysfunction plays a central role in its pathogenesis [166]. The lowered hepatic PC/PE ratio [167] can adversely impair the phospholipid bilayer’s integrity and mitochondrial function, resulting in the perturbation of mitochondrial electron transport, oxidative phosphorylation, and β-oxidation [168].

## 5. MAFLD Preclinical Models

With high association with metabolic diseases, preclinical models for NAFLD are derived from animal models of obesity and/or insulin resistance. Over time, other types of models were developed with other methods, such as chemically induced hepatic insults and genetic models. An ideal preclinical model should meet several of these criteria: (i) closely related to the clinical etiology of the disease, (ii) closely follows the pathogenesis and features of the disease through clinical presentations, such as the presence and severity of steatosis, lobular inflammation, hepatocyte ballooning, and fibrosis, (iii) closely recapitulates the systems-level biology of the disease in humans and (iv) of suitable time-cost effectiveness [169]. Due to the heterogeneity in NAFLD etiology, it has been a major challenge for the field to develop and identify an ideal preclinical model that could closely recapitulate the entire sequelae of NAFLD progression with strong relevance to the human phenotype. This is further highlighted in the change in nomenclature to MAFLD, where the definition of disease encompasses varied combinations of different metabolic disorders. The lack of an ideal model in this heterogeneous and complex disease resulted in NAFLD animal model studies tending to a specific focus on a particular staging or progression of the disease and/or specific causal mechanism. Most established and widely used models have limitations in one or more features of NAFLD in humans. Although such an approach has yielded a significant understanding of disease pathology, there is still an unmet need for the successful development of therapeutics and noninvasive diagnostics. The scale of previous research performed and the lack of progress in these translational areas indicate the dire need for a closely “humanized” MAFLD animal model that is well defined. In this section, we present key features of animal models and their utility and suitability in MAFLD clinical relevance. Furthermore, by summarizing and reviewing the current successes and failures of therapeutic intervention research, we reveal insights into the several shortcomings that could be improved.

First, we categorized model characteristics based on broad categories: (i) metabolic dysregulation-associated, (ii) liver disease-centric, and (iii) pathogenesis-driven. Next, we divide these models into their subcategories: (i) genetic model, (ii) diet-induced model, (ii) specific pathogenesis and/or (iii) various combinations of these types. Finally, we present the models for 3 different criteria: (i) liver phenotype, (ii) metabolic disorder phenotype and (iii) extrahepatic manifestations (Table 1). The liver phenotype is based on histological features that also have similar presentations in humans (Figure 1). Metabolic disorder phenotype describes various systemic disorders of the animal models that are also presented in MAFLD: (i) obesity refers to significantly increased body weight from control, (ii) diabetes, (iii) insulin resistance is tested via glucose tolerance tests, introduced intraperitoneally, intravenously or orally [170], and (iv) dyslipidemia refers to increased plasma cholesterol levels, increased plasma triglycerides, cholesterol, low-density lipoprotein (LDL) and/or decreased high-density lipoprotein (HDL). Due to the cumulative and temporal nature of the animal models, these characteristics are described in terms of the earliest duration of feeding reported in the model, from the start time of the model, that is, the time of diet initiation. In features that are typically present in the model and not associated with a particular onset duration since diet initiation, further model duration will not be recorded (Table 1).

### 5.1. Metabolic Dysregulation Models

#### 5.1.1. Diet-Induced Models

Established in the previous sections, the current paradigm shift in NAFLD toward MAFLD has highlighted the role of metabolic disorder in the disease. This rendered the more preferred use of NAFLD animal models with a metabolic dysregulation basis in studying the disease. Metabolic-dysregulation models usually employ the use of dietary modifications to induce metabolic and liver disease, i.e., diet-induced obesogenic models (DIOs). The most widely used DIO is the High Fat Diet (HFD), where its fat content makes up >60% kcal. However, one key limitation of this model is the slow progression of the disease in rodents. In HFD, rodents must be fed for at least 16 weeks for NASH and hepatocyte ballooning to occur and 60 weeks for progression to HCC. Additionally, severe NASH and fibrosis are not commonly observed. Hence, the field has adapted to modify the macronutrient components in HFD. Specifically, modifications of these parameters still recapitulate the metabolic dysregulation phenotype: (i) type of fats used, e.g., trans-fat, (ii) inclusion of different types of carbohydrates, such as fructose, sucrose and glucose, (iii) addition of cholesterol, and (iv) varying the constituent of components (i)–(iii). To date, there is no consensus on the “optimal” composition of the macronutrient DIO across studies, and the term HFD can be used in various compositions.

The field began to move away from the conventional HFD with the development of the Western Diet (WD) (~40 kcal fat, various % sucrose/fructose, and cholesterol) and the American Lifestyle-Induced Obesity Syndrome (ALIOS) model (45% kcal fat containing 2% trans-fat and fructose in drinking water). Subsequently, the diet was refined, and an amylin liver NASH diet (AMLN) (40% kcal fat containing 18% trans-fat, 20% fructose, 2% cholesterol) was developed. Three years later, it was modified into a Gubra Amylin diet (GAN) as a nontrans-fat substitute due to the ban on trans-fat use in 2018. The GAN used a palm oil substitute as the source of fat [183,184].

With the development of several variations of the HFD, it became clearer that the use of trans fats may not necessarily be relevant to the disease phenotype in the model [185,186]. In a diet composition matched study, the use of a nontrans-fat GAN model was comparable in systemic metabolic dysregulation effects and liver disease progression to that of the AMLN diet (or trans-fat version) [184,187]. Hence, both AMLN [188] and GAN diets [189,190,191] are still used in many clinical-translation interventional studies today. In fact, evidence suggests that the type of fatty acid used in the diet contributes to differential effects in MAFLD [185,192]. Generally, the use of high saturated fatty acids (SFAs) increases the occurrence and severity of NASH development in both animal models and in humans [192,193,194,195] via increased hepatic de novo lipogenesis (DNL) [193], mitochondrial dysfunction, oxidative stress [196], endoplasmic reticulum stress and concomitant hepatocyte injury [82,195]. Consistently, PUFA dietary intake was found to be inversely associated with NAFLD [197], while MUFAs were known to induce protective effects against NASH and HCC [198]. However, a starch-oleate (a major type of MUFA) diet was found to be more similar to WD (high SFA in fat, 40% sucrose, 1.5% cholesterol)-induced NAFLD in driving more severe NAFLD than the starch-palmitate (a major type of SFA) variant [199]. This further indicates the complex and synergistic role of various diet compositions in resulting in differential effects in MAFLD [193,200], consistent with the heterogeneous nature of MAFLD we observed in humans.

Other than the sources of fat used, the use of fructose in DIOs has been highlighted as a major type of dietary carbohydrate that induces a more severe metabolic dysregulation and liver phenotype relevant to humans. In a 2021 systematic review of 3900 models, Im et al. revealed that HFD with high fructose is most relevant to the human disease condition [175,186]. Indeed, this phenomenon is consistent with decades of established clinical studies, showing that high dietary fructose intake is highly associated with NAFLD compared with other saccharides [201,202]. Although fructose metabolism is encompassed within the overall carbohydrate metabolism pathways, particularly leading up to the TCA cycle, the fructose metabolism pathway is distinct and more intricately linked with triglyceride metabolism than glucose metabolism [203]. Principally, the distribution of fructose transporters is highly tissue-specific and is primarily metabolized by the liver through fructokinase-C, which is responsible for driving lipogenic enzymes and hepatic insulin resistance [203,204]. Concomitantly, this drives several MAFLD pathogenic pathways, such as de novo lipogenesis, visceral adiposity, mitochondrial fatty acid oxidation and ER stress [205,206]. Coherently, biochemistry-based computational NAFLD models built on carbohydrate metabolism also revealed that dietary fructose intake has a critical causative role in hepatic steatosis compared to glucose or mixed carbohydrates [207]. Furthermore, dietary fructose also exerts lipogenic effects via gut dysbiosis [205]. In a recent study, it was found that high dietary fructose intake remodels the gut with higher *Proteobacteria* leakage into the liver, causing LPS-induced inflammation and contributing to more severe MAFLD [208]. Together, clinical and mechanistic evidence points toward the involvement of fructose in aggravating MAFLD. Currently, the mode of fructose intake (i.e., solid or liquid form) in DIOs has not been thoroughly studied [186]. Typically, high fat-high fructose DIOs use approximately 20% kcal of fructose in feed or 10–20% in drinking water [175].

The use of an atherogenic basis in the DIO of MAFLD aimed to recapitulate hypercholesterolemia in MAFLD patients and cardiovascular-related comorbidities, as highlighted in the previous sections. This involves the use of cholesterol (0.5–2% kcal) and/or cholic acid in diets such as AMLN/GAN and high-fat high-cholesterol diets. In MAFLD, the deposition of excessive cholesterol directly results in hepatic steatosis and dysregulates cholesterol metabolism [209]. This drives hepatic lipotoxicity, as discussed in the previous sections. Furthermore, compared with HFD, the AMLN/GAN diet is able to induce hepatic steatosis at 12 weeks instead of 16 weeks, NASH by week 20 and fibrosis as early as 26 weeks [185]. The AMLN/GAN diet activates Kupffer cells and hepatic stellate cells, triggering hepatic inflammation and fibrogenesis in worsening NASH. Consistently, clinical cohort studies have also established a dose-dependent protective effect of statins on the severity of MAFLD in patients [209,210,211,212]. The use of cholesterol (1%) with HFD in DIOs was found to aggravate NASH and the severity of MAFLD in multiple parameters with exacerbated weight gain, hepatic steatosis and fibrosis, and serum ALT [213].

Interestingly, recent models exploited inherent differences in crossbred strains in combination with DIO to model NASH. For instance, MS-NASH, previously known as FATZO mice, is a crossbreed of C57Bl/6 and AKR/J strains that specifically selects for dysmetabolic features. MS-NASH mice display ob/ob or db/db-like characteristics of spontaneous obesity and metabolic syndrome, but independent of genetic knockout of leptin or leptin receptors that are not found in humans [214]. The MS-NASH model develops steatosis within 4–8 weeks, although obesity is not obvious at this point. Subsequently, ballooning and NASH developed at 16 weeks, while mild fibrosis developed at 20 weeks. Inherently, MS-NASH mice also develop diabetes [215]. Another such model is the use of isogenic strains of C56Bl/J and S129/V fed a HFD and glucose-fructose water or DIAMOND mice. DIAMOND mice develop steatosis and insulin resistance within 8–16 weeks and hepatocyte balloon and NASH by 24 weeks. F2 stage fibrosis develops at approximately 36 weeks [216]. However, many have cited the peculiar genetic strain, high frequency of HCC and suppression of cholesterol biosynthesis, which deviate from the human condition [171].

Genome-scale network reconstruction revealed that rats and humans share significantly similar metabolic networks, emphasizing their role as model organisms for understanding human diseases [217,218]. Many diet-induced NAFLD/NASH models have been developed in rats, which closely mimic mouse models. Thus far, no original approach has been proposed. While mice and rats have similar responses to dietary challenges, there are differences with respect to susceptibility, severity, and feeding time to develop histological features of NAFLD compared to mice.

In mice, HFD (60% fat) induces strong steatosis with hyperlipidemia and hyperinsulinemia after 10 to 12 weeks of diet [219], while only 7 weeks were needed in rats to observe these features. Long-term HFD feeding induced weak inflammation and ballooning in rats [220]. When Sprague Dawley rats were fed a HFD (71% fat-derived corn oil) or HFD (60% butter-derived fat) for 6–7 weeks, rats on the former diet developed hyperglycemia and macrovesicular steatosis without ballooning or inflammation [221,222,223]. Interestingly, rats on the latter HFD diet developed metabolic syndrome, including obesity, hyperglycemia, and hyperinsulinemia. These observations suggest that fat sources, such as lard, corn oil, coconut oil, fish oil or olive oil, in the HFD have different effects on the development of metabolic syndrome and NAFLD progression. The most pronounced obesity and insulin resistance were observed with lard and olive oil fat [224]. Similar to the HFD mouse models, HFD rat models can replicate the metabolic phenotypes observed in NASH patients; however, inflammation, ballooning and fibrosis were mild or absent.

In many animal models of NAFLD, the addition of cholesterol (0.5–10%) to a HFD promotes steatosis, inflammation, and fibrosis [225]. In combination with cholate (65% fat, 1% cholesterol, 0.25% cholate), Wistar rats exhibited extensive steatosis and inflammation but only mild ballooning after 16 weeks of feeding. However, after 18 weeks of the diet, body weight was lower than that of rats on HDF only [226]. Further modifications were made to the diet by adding either trans-fat as hydrogenated vegetable shortening or fats derived from lard. Rats on the HFHC + trans-fat diet developed only mild fibrosis after 48 weeks [227], whereas animals on the HFHC diet (lard) developed perisinusoidal fibrosis and severe fibrosis at 48 weeks with fibrotic bridges in some rats [228]. Taken together, the quantity of cholesterol and cholate in the diet affects the severity, incidence, and homogeneity of NAFLD but largely fails to induce hepatocyte ballooning and fibrosis. The fat source is another consideration with those derived from lard producing a more pronounced effect. Nevertheless, prolonged feeding of this diet did not accentuate DIO-induced NASH progression.

The increased consumption of sugar-sweetened beverages is closely associated with the development of T2D and obesity, all risk factors for NAFLD [229]. Researchers have used this approach to complement HDF or HDHC to better mimic the etiology of human NASH. Wistar rats that were fed a HFHC diet (49% fat from soybean oil, 1.25% cholesterol, 0.5% cholate, 15% fructose in drinking water) for 7 weeks developed obesity, hepatic steatosis and hypertrophy, with limited inflammation [230]. Sprague Dawley rats on a similar diet with a different fat source (60% fat from lard and corn oil, 10% sucrose in drinking water) displayed obesity, hypertriglyceridemia, hypercholesterolemia, hyperinsulinemia, and inflammatory infiltrate in the liver at week 12 [231]. Other researchers have introduced high levels of various sugars, such as glucose, fructose, and sucrose, in combination with cholesterol and fats into diets [232,233]. Such modifications promoted the presence of metabolic syndrome, and heterogeneous results were also observed in the histological NAS score; however, these HFHS models failed to initiate liver fibrosis.

Rabbits have unique features of lipid metabolism similar to those of humans [234,235] that are not found in rodents, making them a suitable model for studying metabolic diseases. The predominant plasma lipoprotein in mice is HDL. Unlike mice, rabbits are LDL-rich, as in humans. Humans and rabbits have abundant cholesteryl ester transfer protein (CETP) in the plasma, whereas mice do not have an endogenous CETP gene. Finally, apolipoprotein B-48 is present in all apoB-containing particles, such as LDLs and chylomicrons, in mice. In contrast, apolipoprotein B-48 in humans and rabbits is the only specific marker of intestinal chylomicron particles.

Several independent studies have confirmed that high cholesterol in the diet is a key factor in establishing DIO-induced NAFLD in rabbits. New Zealand rabbits fed a similar high-fat high-cholesterol diet (chow diet with 10% lard + 2% cholesterol) for 12 weeks showed significant increases in weight gain. Histological analysis revealed steatosis, hepatic inflammatory cell infiltration, ballooning and perisinusoidal fibrosis [236], recapitulating key features of pediatric NASH. Japanese white rabbits fed either a high-cholesterol diet (HCD, chow diet with 1% cholesterol) or a high-fat high-cholesterol diet (HFCD, chow diet containing 6.7% lard and 1% cholesterol) for 12 weeks exhibited hepatic steatosis compared with the control group. Expectedly, the plasma levels of total cholesterol, triglycerides, and free fatty acids in the HCD and HFCD groups. It was proposed that prolonged endoplasmic reticulum stress (ERS) induced by lipid accumulation causes hepatocyte apoptosis [237]. Using a modified HFCD (chow diet supplemented with 12% corn oil and 0.75% cholesterol), an independent group created a rabbit model of NASH with advanced fibrosis (almost cirrhosis) after 9 months. These rabbits showed high total cholesterol levels in serum and liver tissues in the absence of insulin resistance. Fibrotic septa were found between the central and portal veins [238]. Because this model did not show insulin resistance or obesity, the use of this model may be limited to NAFLD, which is mainly related to hyperlipidemia. To the best of our knowledge, no genetic rabbit model for NAFLD has been developed.

High-fructose diets induce several features of NAFLD in rodents (mice and rats), but similar diets have not been successful in larger animals such as pigs. For example, a high fructose (60%) diet for four weeks to castrated male Danish Landrace-York-Duroc pigs failed to induce steatosis or hepatocellular ballooning in the liver [239]. Similarly, Ossabaw pigs fed a high-fructose diet (20% kcal from fructose, 10.5% kcal from fat, and nil to negligible cholesterol) for 24 weeks had significant weight gain, hypertension, and insulin resistance but showed normal liver histology [240]. Nondiabetic Göttingen Minipigs and minipigs with Type 1 diabetes fed a high fat (43%), high fructose (17.8%), and cholesterol (1–2%) diet for 13 months developed mainly periportal fibrosis and inflammation along with cytoplasmic alterations. Notably, diabetes did not exacerbate the hepatic changes [241]. However, the limited presence of key human-relevant pathological hepatic findings limits their use in preclinical research.

Dietary cholesterol is important in exacerbating NAFLD in pigs [241]. For example, female Ossabaw pigs (starting from 6 months of age) developed hepatocyte ballooning and fibrosis after a 24-week dietary challenge with fructose (18% calories) and fat (43% calories). Nevertheless, no macrovesicular steatosis or lobular inflammation was observed. Supplementing the diet with 2% w/w cholesterol resulted in pigs developing severe NASH with macrovesicular steatosis and lobular inflammation after 16 weeks [242,243]. Ossabaw pigs had metabolic syndrome and abnormal liver histology on an atherogenic diet (20% kcal from fructose, 46% kcal from fat, 2% cholesterol by weight) with significant microvesicular steatosis and fatty Kupffer cells but no ballooning or fibrosis.

The most common NAFLD pig model is based on Ossabaw pigs fed a modified fructose-based atherogenic diet (46% kcal from fat, 17.8% kcal from fructose, 2% cholesterol, and reduced choline). This model developed severe metabolic syndrome and abnormal liver marked by macrovesicular and microvesicular steatosis. Hepatocyte ballooning was observed at 8 weeks, which progressed to extensive ballooning (>90% hepatocytes) at week 16 and evidence of moderate hepatic fibrosis [243]. Compared with controls, this modified atherogenic diet had significantly lower serum adiponectin but higher leptin and TNF levels and higher hepatic triglyceride and malondialdehyde levels. It is worth noting that the fat calories were produced by a mixture of hydrogenated soybean oil (only in the atherogenic diet), coconut oil, and lard, hinting that the type of fats may also play a role in NAFLD.

Overall, the inclusion of fructose and cholesterol in diets such as GAN and other nonstandardized models have been reported to mimic the disease presentations and transcriptomic profiles as in humans, cementing its widespread use in interventional and mechanistic MAFLD studies in recent years [183,185,244,245,246].

#### 5.1.2. Genetic and DIO Combination Models

Genetic obesogenic models have also been used to recapitulate MAFLD [247]. The commonly used leptin deficient (ob/ob) or leptin receptor deficient/leptin resistant (db/db) background has been commonly used to replicate obesity involvement in MAFLD. Leptin is a satiety-related hormone that regulates the appetite of the mouse. Genetic models ob/ob and db/db are unable to control their appetite and consume excessive or hyperphagic food, resulting in obesity [248] and type II diabetes [249]. In the long term (>16 weeks) of feeding a chow diet, ob/ob or db/db models develop mild hepatic simple steatosis (Table 1). These models are often utilized to study early MAFLD stages, such as mechanisms in steatosis onset (>12 weeks). Moreover, to reduce the duration of disease models, it is a very common practice that DIO is used in combination with ob/ob and db/db backgrounds to promote disease progression [183]. However, the controversy in using ob/ob or db/db models is due to the lack of leptin gene variants in humans [169]. Hence, although the genetic model does develop obesity, insulin resistance and early MAFLD, it does not contribute to the pathogenesis of MAFLD. The use of this model requires critical assessment of the relevancy in the research question involved.

Genetic models with an atherosclerosis basis are also employed to recapitulate the cardiovascular complications in MAFLD. For instance, apolipoprotein E (ApoE) deficiency is a common genetic feature in developing CVD due to the inability to maintain cholesterol and triglyceride homeostasis, resulting in dyslipidemia and the formation of atherosclerotic plaques in humans. Hence, APOE−/− models were first developed to investigate APOE involvement in CVD. Interestingly, it has been recently revealed that an APOE variant is also associated with higher NAFLD occurrence [250], while a cohort study indicated that APOE ε3/ε3 genotypes increased the patient risk of developing NAFLD [251]. In MAFLD studies using ApoE−/− models, ApoE−/− inherently develops simple steatosis at approximately 12 weeks of age through derangements of hepatic lipid metabolism and hepatic lobular inflammation with NASH and F1 fibrosis by week 52. The majority of the studies used a HFD [252,253] or an atherogenic diet (including 0.1–2% cholesterol) [254] to exacerbate diet-induced MAFLD [255,256,257]. Similarly, the Ldlr−/− model is also used in conjunction with DIO to induce MAFLD and associated CVD [258]. With high fat-, high fructose-, and high cholesterol (~0.05% w/w) diet feeding for 28 weeks, severe NASH and fibrosis (F3 stage) occurred simultaneously with atherosclerotic lesions along the aorta [258]. Other groups have developed an ApoE-LDLR double-deficient model with high fat (35%) and high cholesterol (5%) feeding that resulted in HCC development and defective hepatic vasculature at 35 weeks [259,260].

Previously, the use of KK-A^y^ models with HFD was adopted to model T2DM-MAFLD. KK-A^y^ is a transgenic model that is generated from the cross of diabetic KK and lethal yellow agouti (A^y^) genotypes resulting in systemic defects in the PTEN, PPAR-α, AOX, and MAT1A pathways [173]. This results in a similar metabolic dysregulation phenotype compared with NAFLD patients [261]. The lack of severe and significant steatosis and NASH development resulted in poor usage of this model. However, Sakuma et al. recently developed a combination model utilizing KK-A^y^ mice with high fat, fructose and cholesterol with a cholic acid supplementation diet. They reported metabolic dysregulation involving obesity, insulin resistance and hepatic dyslipidemia together with NASH at 4 weeks and fibrosis at 12 weeks of diet [262]. As a KK-A^y^ is conventionally a T2DM model, this study not only provides a means to study the link between T2DM-MAFLD disorder but also highlights the necessity and differential effects that diets can have on metabolic disorders.

*SREBP-1* gene polymorphisms are associated with obesity, severe insulin resistance, and T2DM in humans [263,264]. Transgenic spontaneously hypertensive rats (SHRs) overexpressing SREBP-1 are nonobese but develop hypertension, hyperglycemia, hyperinsulinemia, and fatty liver [265]. NAFLD progression is age dependent. At 2 months of age, centrilobular steatosis was present without inflammation. However, focal hepatocyte necrosis and inflammatory infiltrate were found after 16 months [266].

Otsuka Long-Evans Tokushima fatty rats (OLETFs) were obtained by selectively breeding a Long-Evans rat colony with a diabetic phenotype, including polydipsia and obesity [267]. OLETF rats lacked the cholecystokinin-1 receptor due to a deletion of the promoter region and the first 2 exons of the gene. The OLETF rats spontaneously developed liver steatosis when fed a standard chow diet [268]. At 22–38 weeks, micro- and macrovesicular steatosis and hepatocyte ballooning were evident in their livers. However, no fibrosis was observed even until 42 weeks. When OLETF rats were fed with MCDD, they developed hepatic lobular inflammation and fibrosis [269,270].

Prague hereditary hypercholesterolemic rats, crossbred from Wistar rats, are a model for polygenic hypercholesterolemia induced by dietary cholesterol (2%). Cholesterol began to accumulate in the liver after one day and progressed with feeding. This cholesterol accumulation is accompanied by triglyceride accumulation, culminating in fatty liver [271,272]. However, this model of fatty liver is not widely used, and thus little information is reported about NAFLD progression to NASH and fibrosis.

The most commonly used genetic model of NAFLD in rats is the obese Zucker rats, also known as *fa/fa* rats or ZFR. The ZFR harbors a mutation in the leptin receptor, which decreases the affinity for leptin [273]. As a result, ZFR develops severe obesity and insulin resistance [274]. Macrovesicular or microvesicular steatosis spontaneously developed in ZFR but did not progress to NASH [275]. When fed any NASH-inducing diet, such as HFD or MCDD, ZFR developed more severe micro- and macrovesicular steatosis and NASH [276].

Given the polygenic background of NAFLD, the limited monogenetic rat models do not reflect the etiopathogenesis of human NAFLD. Nonetheless, they are valuable for understanding a particular stage or event of the disease.

The physiology and metabolism of humans and pigs are highly similar. Previous studies using rodents also underscored an important role of leptin in driving the formation of liver fibrosis. Consequently, a leptin-deficient pig (*LEPTIN^−/−^*) was generated using the zinc finger nuclease approach [277]. *Unlike* leptin deficiency in rodents, which does not result in hepatic fibrosis, *LEPTIN^−/−^* pigs developed the phenotypic features of fatty liver, NASH, and hepatic fibrosis with age.

*LEPTIN^−/−^* pigs exhibited significantly elevated levels of glucose, triglycerides, total cholesterol, and LDL at 12 months and 18 months, while HDL was reduced in the serum. At 0–6 months, there were no histological differences between *LEPTIN^−/−^* and wild-type livers. By 6–12 months, lipid deposition and hepatocyte steatosis in *LEPTIN^−/−^* pig livers increased. At 12–22 months of age, the livers of these mutant pigs revealed features of early-stage fatty liver disease, with obvious balloon degeneration and vacuolated necrosis at the middle and late stages. In addition, immune cells infiltrated the hepatic lobule portal area and between the hepatic parenchymal cells of hepatic lobules. Finally, between 22 and 35 months of age, the *LEPTIN^−/−^* pigs showed a high degree of hepatomegaly with fibrosis. These results suggest that *LEPTIN^−/−^* pigs developed middle or advanced stages of fibrosis but not cirrhosis.

#### 5.1.3. Summary Remarks for Metabolic-Dysregulation Models

With the shift toward a more metabolic-dysregulation focused MAFLD, models exhibiting metabolic disorder are increasingly gaining its foothold as the definitive model for investigating MAFLD. However, due to the complexity of metabolic disorders, there has not been a model that can fully recapitulate the disease features of humans. The use of varying compositions of diets as well as the use of purified or complex food sources results in different effects on animal models. Hence, it is important for researchers to report the type of diets they are using in terms of % kcal, w/w or w/v, depending on the macronutrient. This practice has not been well established previously, and there are many groups using nonrepresentative or nonself-explanatory model names (e.g., HFD naming but also including cholesterol and fructose or WD naming but not reporting macronutrient composition). Our understanding of MAFLD often points to the heterogeneity of the disease. The lack of a standardized diet further accents this feature, among the other evidence presented in this section. Although this encourages diets of different macronutrients to be explored, perhaps the field requires a more systematic approach in designing the most appropriate diets for the specific research questions.

The MAFLD paradigm also emphasized other extrahepatic malignancies (Section 4). Hence, in animal studies, it is ideal that such disease presentations will occur, and it will be increasingly important to interrogate the underlying mechanism in interorgan crosstalk. To circumvent the problem of not having an ideal model, a different genetic + DIO combination model can be adopted. For example, ApoE-deficient or LDLR-deficient models are coupled with HFD for CVD-MAFLD interplay, while KK-Ay-high-fat-high-fructose-high cholesterol with cholic acid models can be used for T2DM-MAFLD [262]. As mentioned in Section 4, stratification of MAFLD patients into various sub-MAFLD definitions, such as T2DM, obesity or metabolic syndrome, also yielded differential prognoses. Hence, such models are required to be further established and interrogated for mechanistic and interventional directions to further the understanding of MAFLD.

### 5.2. Liver Disease-Centric Models

In this subsection, animal models focusing on exacerbating hepatic insults are reviewed and discussed. Often, these models do not present metabolic derangements. However, they are commonly used to understand the mechanisms driving liver disease progression.

#### 5.2.1. Diet-Induced Models (Nutrient Depletion)

To exacerbate liver injury, micronutrient-deficient diets were used. A popular diet-induced model is a methionine- and choline-deficient diet (MCD). In this model, severe steatosis, NASH and fibrosis occurred after 8 weeks of feeding (Table 1). This is due to impaired beta-oxidation and lipoprotein biosynthesis pathways required by the 2 essential nutrients [278]. However, a huge disadvantage in using MCD-fed models is that they do not exhibit insulin resistance and obesity [213], a symptom found in patients’ MAFLD. Furthermore, MCD models usually experience weight loss as a result of lower caloric intake. Together, these findings indicate poor human translatability of systemic disorders in MAFLD present clinically. To induce metabolic dysregulation in MCD models to recapitulate late MAFLD, various groups have combined HFD diets with methionine and choline nutrient deficiency. Interestingly, despite 60% kcal fats in MCD, these rodents continue to lose weight, suggesting that depletion of these essential amino acids contributes to a different underlying disease mechanism compared with the common dyslipidemia-associated MAFLD. Coherently, inclusion of trace amounts (~0.1–0.15% (w/w)) of methionine and/or ~0.06% choline in the diets slowed NASH progression but resulted in obesity, insulin resistance and dyslipidemia at 23 weeks [279]. Recently, Wang et al. [278] proposed the key role of a disordered methionine cycle in driving NAFLD progression during early steatosis stages. This could possibly provide the link that we observe in MCD-aggravated liver disease, independent of metabolic dysregulation [278].

In the same vein, a choline-deficient L-amino acid controlled (CDAA) diet is a variant of the MCD. The use of CDAA was developed to mimic the aggravated hepatic insults observed in MCD. With the inclusion of methionine and controlled L-amino acid composition, rodent models did not exhibit weight loss [280]. However, similar to MCD, systemic metabolic dysregulation was not found [280,281]. Typically, CDAA is also used under a HFD background (CDAA-HFD) with 40–60% kcal, which accelerates the disease progression more rapidly together with induced expression of fibrogenic pathways [180]. However, similar to MCD and CDAA, CDAA-HFD also does not exhibit metabolic dysregulation [180].

Sprague Dawley rats fed an MCD diet for 6–8 weeks developed severe steatosis with mild inflammation and no ballooning or fibrosis [282,283]. Similar observations were reported for three common rat species, Sprague Dawley, Long-Evans and Wistar rats, fed the MCDD for 13 weeks [284,285]. However, Wistar rats had more exacerbated liver lipid levels, serum ALT levels and liver mass:body mass ratios than Long-Evans and Sprague–Dawley rats, suggesting that Wistar rats are more prone to developing steatosis. The CDAA diet is similar to the MCD diet but has less methionine (0.1%). Wistar rats on CDAA for 6–12 weeks developed severe steatosis with inflammation but lacked ballooning phenotypes [286]. The livers of these rats displayed elevated ROS after one week of feeding and impaired mitochondrial function at 16 weeks [287]. Chronic feeding (40 weeks) resulted in severe fibrosis and, interestingly, adenoma that progressed to HCC in 50% of the cases [288,289]. While these DIO models mimic the histopathological features of NAFLD, the absence of metabolic syndrome and unusual amino acid deficiency that is uncommon in human diets limit their relevance when the etiology of NAFLD is considered [290].

#### 5.2.2. Chemically Induced Models

Diethylnitrosamine (DEN) is a carcinogen that causes hepatic injury leading to fibrosis, cirrhosis and HCC by forming mutagenic DNA adducts [291]. In MAFLD-HCC models, DEN is injected intraperitoneally into rodent models fed a HFD to induce HCC. Interestingly, it was reported that under a HFD background, HCC, inflammation, and fibrosis induced by DEN are exacerbated [292,293]. However, in these models, NASH, fibrosis is absent and is hence unable to capture the sequelae of MAFLD progression and consequently NASH-driven HCC [294].

Another commonly used method to drive liver disease to HCC is by administering carbon tetrachloride (CCl4). CCl4 acts as a toxin that drives hepatic injury through the cytochrome P450 superfamily of monooxygenases (CYP family) into trichloromethyl radicals. The presence of volatile radicals reacts with various biomolecules—nucleic acids, proteins and lipids—resulting in DNA damage and defective metabolic pathways driving HCC [171,295].

The combination of WD (~40% kcal fat, ~40% carbohydrate) with weekly CCl4 dosage is typically used to model NASH-driven HCC. Compared with no CCl4 administration, NASH progression is accelerated, F3 stage fibrosis is observed by weeks 8–12, and HCC develops by week 24 [173,214]. Several studies have highlighted the dosage-dependent effect of CCl4 administration on hepatic injury [214,296]. Thus, the use of this model requires critical relevancy of the CCl4 dosage regime and specific research scope.

#### 5.2.3. Summary Remarks for Liver Disease-Centric Models

Together, these models target the later stages of MAFLD progression. In MCD, CDAA and CDAA-HFD, the induction of chronic hepatocyte stress and chronic inflammation resulting in fibrogenic processes renders these models relevant in developing focused studies and shortened timelines to investigate mechanisms involved in late-stage liver disease (Table 1). On the other hand, chemical insults such as the use of CCl4 and DEN drive the disease into end-stage cirrhosis and HCC (Table 1). However, this would not fully recapitulate MAFLD per se, without metabolic derangement and/or extrahepatic manifestation in CVD or CKD. Hence, these models can only be classified or used in liver disease-specific investigations but not MAFLD, where the lack of systemic dysregulation would fail to model the interorgan crosstalk in the disease. Nonetheless, interventional studies could be performed to identify therapeutics that mitigate hepatic complications. These approaches can then be used on a metabolic-dysregulation model to investigate the systemic effects of the drug in a more MAFLD-relevant model, or vice versa. One example of such an application is liraglutide, a GLP-1R agonist that has been approved for T2DM [297]. The drug was repurposed and applied in an MCD model to investigate its effects on MAFLD progression, independent of its established metabolic effects [297], providing further evidence on its use clinically. In the future, combination therapeutics can also be developed by targeting both liver disease progression and metabolic dysregulation. Despite this, the caveat remains that MAFLD-associated cirrhosis and HCC may not have the same underlying biology as those that are chemically induced with carcinogens. Nonetheless, a methionine- and choline-deficient diet (MCD) is still widely used today as an interventional study against severe liver disease [298].

### 5.3. Pathogenesis-Driven Models

In this section, major animal models that were developed on the basis of a specific etiological/pathogenic pathway known in MAFLD are presented.

#### 5.3.1. From Genetic Studies

Previously, genetic studies of NAFLD revealed highly associated genes that correlate with the occurrence of NASH. In particular, *PNPLA3 rs738409* is among the few single nucleotide polymorphisms (SNPs) recurrently highlighted in independent clinical studies [299,300] The mechanism of *PNPLA3* in MAFLD disease contribution is further explored in Section 5.2. Consequently, animal models with these variants were developed. In a landmark interventional study of the PNPLA3 gene, a human-relevant PNPLA3-I148M SNP variant was introduced into C57BL/6 mice through homologous recombination to obtain heterozygous (PNPLA3–148I/M) and homozygous PNPLA3–148M/M) mice, which were subsequently fed a modified DIO (in this case, a high sucrose diet) to induce MAFLD [301]. The study provided proof-of-concept in using an antisense oligonucleotide (ASO) against PNPLA3-I148M to ameliorate hepatic steatosis and NASH development and is currently in clinical trials (NCT04142424, NCT04483947) for patients who are homozygous for the variant [302]. Despite this, it is interesting to note that PNPLA3-I148M variant mouse models do not exhibit any physiological differences when fed DIO or control unless the PNPLA3-I148M variant is overexpressed, unlike in MAFLD patients [171]. Furthermore, *PNPLA3* variants in animal models do not exhibit insulin resistance despite high sucrose or high fat feeding [303,304,305].

#### 5.3.2. ER Stress

With the concept of studying ER stress in NASH and the failure to simulate NASH-driven HCC using HFD + DEN, Nakagawa et al. developed the MUP-urokinase plasminogen activator (uPA) transgenic mouse fed a HFD model [306]. In this model, uPA is specifically overexpressed in hepatocytes under the control of the promoter for major urinary protein (MUP), driving transient ER stress. ER stress is an established pathogenesis of MAFLD promoted by dyslipidemia and metabolic dysregulation resulting in hepatoxicity (Section 5.3). MUP-uPA was reported to exhibit human-like features of NASH—insulin resistance, obesity, hepatocyte ballooning, inflammation, and advanced fibrosis by 16 weeks of HFD feeding. Eighty-five percent of these mice with NASH develop HCC after 32 weeks [173,307]. Furthermore, the MUP-uPA model was able to recapitulate p62 accumulation in HCC development and several of the gene signatures associated with human NASH [308]. However, due to the use of a transgenic model that does not follow patient NASH etiology, it has not been prevalently used in NASH studies until now.

#### 5.3.3. Immune System and Inflammation in MAFLD

As discussed in the previous sections, low-level chronic inflammation is a crucial factor driving MAFLD across MAFLD disease progression. Hence, many models focusing on the immune system have been developed to investigate the inflammatory response. The first such study involved the use of administering LPS into mice in conjunction with HFD, which resulted in the development of the initial “two-hit hypothesis” entrenching the necessary role for inflammation in MAFLD [309]. Today, the immune profile of MAFLD patients is much more complex [310,311,312]. In particular, there are complex interactions between innate and adaptive immune responses, gut dysbiosis, and differential immune profiles across various stages of MAFLD progression [315,316,318]. Mechanistic studies into various immunological responses broadly involved immune cell-deficient, adoptive transfer models and/or humanized immune system models in combination with DIO. The immune response is highly complex, dynamic and heterogeneous. For instance, dendritic cells (DCs) are professional antigen-presenting cells that act as bridges between the innate immune response and activation of the adaptive immune response. Consistently, in a DIO (54% kcal fat) model with knockout of CD40 expression in CD11c+ (CD40^fl/fl^ CD11c^cre^), where CD40 is a costimulatory molecule that is involved in T-cell activation, hepatic inflammation was ameliorated [313]. However, adoptive transfer of CD103+ conventional DCs (cDCs) into a dendritic cell deficient (Batf3^−/−^) protected the progression of simple steatosis to inflammatory NASH stages [314,315].

The heterogeneity within DC subsets has differential roles in lipid accumulation, mounting proinflammatory responses and both anti- and pro-fibrogenic responses [314]. The involvement of DCs in lipid metabolism within systemic and hepatic contexts emphasizes the important use of an appropriate MAFLD disease model due to the dynamic role of immune cells not only in mounting inflammation but also in other metabolic disorders. These insights were revealed through the combination of various models in manipulating the immune system in MAFLD models. Consistently, a seminal study on the role of lymphocytes in MAFLD found that mice deficient in T and NK cells did not develop NAFLD [316]. Depletion of T cells, in particular, ameliorated insulin resistance, weight gain and adiposity in DIO models [310]. In a humanized intrahepatic CD4+ T-cell model of DIO, it was found that human memory CD4+ T cells are important in driving liver inflammation and fibrosis [317]. However, the humanized immune system in mice is tedious and complex and requires further validation.

#### 5.3.4. Gut Dysbiosis in MAFLD

In recent years, there has been increasing attention in attempts to elucidate the dynamic role of gut dysbiosis in MAFLD pathogenesis using animal models. In a seminal work relating gut dysbiosis and NAFLD, Le Roy et al. (2013) demonstrated that transplantation of fecal microbiomes from DIO mice into germ-free mice conferred metabolic syndrome and an increased hepatosteatosis-associated transcriptomic profile, indicating the pivotal role of the gut microbiome in driving NAFLD [131]. Indeed, microbiome transplantation is a widely used preclinical animal model approach in MAFLD gut dysbiosis studies [318]. Microbiome transplantation allows for the repopulation and manipulation of the gut microbiome in animal models to investigate the physiological effects of the hypothesized composition. In MAFLD animal models, microbiome transplantation experiments are typically performed together in DIO or nutrient depleted MAFLD-induced mice with additional approaches to interrogate gut microbial composition through metagenomics or 16S-rDNA amplicon sequencing analyses [319].

Clearly, mechanistic studies involving gut dysbiosis typically also require the use of gnotobiotic animal models. In this case, rodent models are usually the species of choice due to practical reasons in terms of caging facilities and the ease of manipulation [320,321]. To achieve a controlled gut microbiome in gnotobiotic animals, the incumbent host microbiome is eliminated using either germ-free mice [321] or antibiotic-induced microbiome depleted mice (AIMD). Subsequently, microbiome transplantation can be performed to repopulate the gut of these animal models for mechanistic microbiome knock-in studies [322].

These technologies enable not only characterization studies but also interventional and mechanistic studies on the gut-liver axis and MAFLD. For instance, interventional transplantation of metabolic disease-associated *Akkermansia muciniphila* into HFC DIO mice was found to reverse MAFLD in the liver through *Akkermansia*-mediated maintenance of gut barrier integrity and an increase in L-aspartate transport to the liver [323]. In another recent study, the use of a gut microbiome-modulating ileal bile acid transport inhibitor (IBATi) drug was found to alter the gut microbiome in HFD DIO mice, causing amelioration of MAFLD. The investigators transplanted IBATi-treated mouse fecal microbiomes into AIMD mice and revealed that restoration of a-diversity and *Firmicutes:Bacteroidetes* was involved in the amelioration of disease-related metabolic dysregulation in MAFLD mice [324]. In recent times, such combinatorial microbiome-related and animal model approaches have been adopted for a multitude of interventional factors ranging from small-molecule drugs [325] and defined dietary supplements or compounds [326,327,328,329] to herbal extracts [330,331,332].

With the intricate interactions between the gut microbiome and host immune response, many recent studies have dwelled deeper to unravel complex interorgan mechanisms using MAFLD animal models. In a recent study, Kawano et al. (2022) elegantly put together a series of experiments involving HFD DIO and dietary sugar, CD4+ T cells and Th17 ablated mice and probiotics-mediation enrichment of segmented filamentous bacteria (SFB) and demonstrated that the remodeling of gut microbiome-immune related Th17 by dietary sugars drives metabolic dysregulation in mice [333]. Expectedly, such complex animal model designs will continue to innovate and be published increasingly over time in the translational MAFLD field.

#### 5.3.5. Summary Remarks for Pathogenesis-Driven Models

In genetic models developed from the basis of population genomics studies such as *PNPLA3*, complex mechanisms underlying allelic risk must be considered. In this case, the initial belief that the PNPLA3-I148M variant only results in the loss of function in lipid metabolism did not explain the underlying biology [303]. PNPLA3-KO models did not generate a NASH phenotype, and thus, further knock-in experiments of PNPLA3-I148M reveal that an additional gain-of-function mechanism causes a lack of ubiquitination in PNPLA3-I148M resulting in a “gain-of-function” in reducing protein turnover [303,334]. This indicates that in genetics-based studies, caution must be taken to elucidate the molecular basis behind a driver gene—the mechanism in animal models may not necessarily follow the phenomena observed in humans [303].

Although the field has advanced with the introduction of various models to mimic and manipulate gut microbiome and inflammatory responses, these are all highly influenced by the underlying diet background (DIO). The heterogeneity of the disease pathogenesis even within the various animal models and inflammatory responses is not trivial and could explain the seemingly controversial mechanisms we observe across various studies [72,335]. Currently, immunological mechanisms summarized by several reviews are based on MAFLD models of various metabolic backgrounds, e.g., DIO and MUP-uPA. However, it is unknown whether the immunometabolic landscape is aligned across these models and relevant to humans. Likewise, it is crucial to investigate the translatability of animal models with that of the human condition [336,337]. Hence, the standardization, humanization and proper characterization of animal models are highly important. It is currently still unclear whether these differences in models are biologically significant in MAFLD.

## 6. Perspectives

The development and progression of MAFLD involve gene–environment interactions and multiorgan communication associated with extrahepatic presentations such as insulin resistance, cardiovascular and kidney problems, and even intestinal microbial changes. This new nomenclature highlights the importance of disrupted metabolism in liver disease development. Clinically, it reclassifies patients with impaired metabolism rather than a set of exclusion criteria to rule out alternative disease etiology. Not only does it credit early-stage disease development, it also emphasizes a continuous spectrum with progressive disease worsening [25]. While the need to understand more about the disease in a time-dependent context, this task is confounded by the dormant nature of the disease progression, having nearly no symptoms in the initial stages of the disease. Furthermore, NASH development requires histopathology analysis and repeated biopsies, limiting eligible study participants to only those in need of the procedure [338]. As such, patients often seek medical help after significant disease progression, delaying treatment and study recruitment, leaving disease pathogenesis poorly understood.

Current diet-induced NAFLD models have provided important insights into the pathogenesis of MAFLD. However, prolonged feeding to develop limited human NASH features, including metabolic aberrations, deters longitudinal studies required to reveal disease progression and temporal physiological changes. For example, C57BL/6 mice on a HFD showed only minor signs of inflammation and fibrosis even after prolonged feeding for up to 50 weeks [219]. While the MCD model overcomes the lack of severe hepatic fibrosis often observed in many preclinical models, these mice showed no sign of insulin resistance and suffered weight loss and a reduction in liver weight [339,340]. The DIAMOND model is based on isogenic C57BL/6xS129S1/svlmJ mice and has been shown to recapitulate key physiological and metabolic features of human NASH. However, a limitation of this model is the high frequency of hepatocarcinoma and suppression of cholesterol synthesis, which is substantially different from the human situation [171,216].

There are plenty of ongoing discussion regarding NAFLD and MAFLD. While they share significant overlaps, there are also clear differences in the prevalence and risk factors between MAFLD and NAFLD [37]. MAFLD places greater emphasis on concomitant metabolic diseases. We share two perspectives that were largely ignored in this field. These perspectives, while relevant to NAFLD, emphasize physiology, and the development of metabolic syndrome will aid in navigating NAFLD to the MAFLD paradigm.

### 6.1. Comparison with the Right Control Diet

Only approximately 20% of diet-based research utilizes properly matched control diets to their treatment counterparts [341,342]. Standard regular chow, contrary to being “standard”, is composed of unrefined ingredients from wheat, cereal, and animal byproducts. As a control maintenance diet, it is enticing due to its widespread adoption and low cost. However, when comparing the effects of a defined MAFLD-inducing diet such as HDF with regular chow, it does little to address experimental requirements and confound experiments due to compositional inconsistencies [343]. Regular chow diets are vulnerable to supply chain variation, the location and conditions of harvest and processing techniques, which have a huge impact on fiber content [344].

The gut microbiota has emerged as an important environmental factor influencing the pathogenesis of MAFLD through the gut-liver axis [345]. Manipulation of the gut microbiome is sufficient to initiate a phenotypic change and implicate overall disease progression. For example, high fat in various diets has been well reported to induce obesity and other metabolic diseases. However, carefully controlled studies also suggest that fibers in the diet, in addition to the level of fat, play a key role in the development of metabolic disease [346,347]. Regular chow is made up of 20% total fiber, a mix of soluble and insoluble fiber. In contrast, most purified diets historically are composed of approximately 5% total fiber that comprises insoluble fiber in the form of cellulose. Gut bacteria can ferment soluble fiber, whereas insoluble fiber is largely not fermentable. The liver receives more than half of its blood circulation from the splanchnic district, making it especially vulnerable to gut-derived toxins and metabolites and presenting itself as a first line of defense against intestinal pathogens [348].

Refined diets empower researchers with more control over their experiments, ensuring that changes in metabolic parameters are due to experimental variables and not inconsistencies in diet composition. Hence, prior research attributing phenotypic and mechanistic differences in animals based on grain-based diet comparison could have unintentional effects on the parameters studied. With a growing interest in gut microbiota that are deeply impacted by fiber types and composition, such differences are critical to metabolic research, including MAFLD [349].

### 6.2. Keeping It Warm

The standard housing temperature of 22–25 °C causes mild cold stress, which results in major deviations from human metabolism. A cooler environment results in additional energy expenditure to maintain core body temperature and changes to heart rate and blood pressure, which greatly augments mouse metabolism as they switch to non-shivering thermogenesis and elevate brown adipose tissue production [350,351]. Behavioral changes, such as nesting and huddling, further contribute to the list of confounders, as the number of mice in the cage and bedding material will affect overall metabolic changes. Thermoneutral housing aligns mouse daily energy expenditure to be near human resting values [352]. Recently, the use of thermoneutral housing has been shown to exacerbate liver disease progression while eliminating gender differences in the development of disease. Despite accelerated progression to NASH, these mice did not develop hepatic fibrosis, thus underscoring the importance of diet composition [353].

MAFLD exacerbation at thermoneutrality was conserved across multiple mouse strains and was associated with augmented intestinal permeability, an altered microbiome and activation of inflammatory pathways that are associated with the disease in humans [353]. Housing mice at 22 °C increased the *Firmicutes:Bacteroidetes* ratio compared to when mice were housed at 30 °C. Cool housing also leads to increased production of bile acids, which in turn alter the microbiome to promote thermogenesis in lab mice [354]. Inflammation, a key player in NASH progression, is also significantly affected, with a suppressed immune response caused by changes in the sympathetic nervous system. A key mechanism for cool adaptation in mice is the elevation of sympathetic drive, which increases the heart rate and turnover in adipose depots [355]. Chronic activation of the sympathetic nervous system results in suppression of the immune system as energy is directed from the immune system to heat generation. Thermoneutral housing depletes the Gram-negative microbiota, depletes TLR4 in hematopoietic cells and inactivates the IL-17 axis, resulting in altered immune responsiveness and protection from MAFLD progression [354].

A better model should exhibit metabolic abnormalities and a full spectrum of MAFLD progression with histological features such as hepatocyte ballooning and fibrosis. A shorter progression time can also serve to accelerate research and reduce age-related confounders. A positive response to lifestyle changes, similarly, observed in humans, will enhance its physiological relevance to human MAFLD. These two viewpoints should be considered in diet-based studies of MAFLD and urge researchers to refine murine models of MAFLD to increase their translational capacity.

### 6.3. Landscape of Preclinical Models in the NAFLD-to-MAFLD Transition

**Figure 4:**
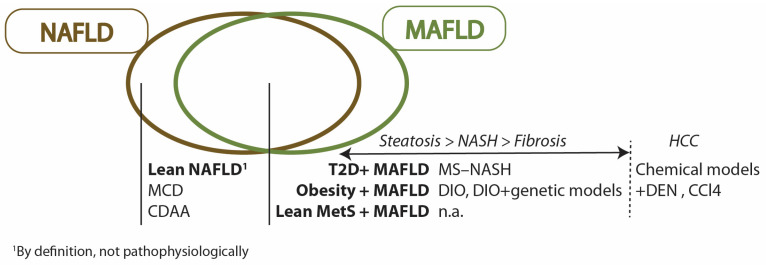
Preclinical MAFLD models in the NAFLD-MAFLD landscape.

Generally, DIO or DIO-genetic models lie in the overlapping definitions of NAFLD+MAFLD+. This is expected, given that most DIO models are generated from excessive feeding of dietary nutrients resulting in systemic metabolic derangements. As discussed in Section 3, emphasis of metabolic dysregulation in MAFLD definitions and its subcategories (i.e., T2DM, obesity, metabolic syndrome) has suggested differential outcomes in fatty liver disease progression and extrahepatic comorbidities. Hence, we illustrated the utility of these preclinical models in stratifying MAFLD subgroups (Figure 4).

On the other hand, FLD-progression nutrient depletion models (e.g., MCD, CDAA) [356] do not adhere to MAFLD definitions due to the lack of obesity, T2DM or metabolic syndrome presentations. However, they do fall into NAFLD definitions with the presence of hepatic steatosis and not secondary causes of liver disease. Despite this, these models presented more aggressive FLD progression, indicating that the pathological mechanism of choline deficiency disease presentations is not systemic-related. Although MCD models reduced body weight, plasma triglycerides, cholesterol, and glucose levels and improved HOMA-IR [356], in contrast to metabolic NAFLD, MCD and CDAA models are considered lean NAFLD given the latest guidelines [357]. Despite this, the extent to which MCD and CDAA recapitulate the clinical presentations in lean NAFLD is arguable. Compared with non-NAFLD lean subjects, metabolic parameters such as serum TG, LDL, and HOMA-IR are increased, indicating metabolic syndrome features, dyslipidemia and insulin resistance [358,359]. Furthermore, metabolic dysfunction and T2DM were identified as major risk factors for driving hepatic fibrosis in lean NAFLD [50,359]. As such, although MCD and CDAA accelerate FLD progression and are considered lean NAFLD by definition, they are unable to fully recapitulate lean NAFLD [182,359]. Future studies using such models must be scrutinized closely for their relevance to specific NASH research questions. This could also possibly explain the failure of clinical drugs that were previously validated by nutrient-depleted NAFLD models [297].

Overall, the redefinition of NAFLD to MAFLD refocuses the translational MAFLD field toward systemic metabolic dysregulation, which includes lean NAFLD and all other subtypes of MAFLD. Furthermore, the differential outcomes of the different subtypes suggest an underlying difference in pathogenesis and disease drivers among the T2D + MAFLD, obesity + MAFLD and MetS + MAFLD subgroups. Hence, the shift away from NAFLD is a step toward delineating the disease into its various metabolic disorder manifestations. This calls for researchers to be more sensitive in disease stratification even at the level of preclinical MAFLD basic research.

### 6.4. Applications of Preclinical Models in Basic and Translational Research

To date, there is no FDA-approved drug for MAFLD. Due to the recency in the change of nomenclature, the disease is still considered NAFLD in clinical trials. As such, discussions on the state of therapeutic development and references to clinical trials will consistently use the NAFLD definition. This failure of therapeutic development has been largely attributed to several reasons. First, the heterogeneity of NAFLD patients affects disease outcomes and presentations [360,361]. The second is the misalignment of the liver disease progression stage of the recruited NAFLD patient cohort [360,362]. Third, there is a lack of practical and meaningful (surrogate) endpoints [360,363].

From a preclinical research perspective, more research needs to be done to address some of the issues in the therapeutic development pipeline. Given the changing landscape of NAFLD-to-MAFLD transitions, greater specificity is required by researchers to align the metabolic subgroupings of these animal models [364]. With the increased emphasis on metabolic dysregulation, metabolic parameters of animal models must be characterized comprehensively. This includes commonly investigated parameters relating to dyslipidemia (serum TG, FFA, cholesterol) content, insulin or glucose tolerance tests, body weight and adiposity. Furthermore, extensive comparative analyses at the metabolic, multiomics and histological levels are required to ensure relevancy in human conditions [365]. This is incongruent with many MAFLD multiomics studies published in recent years [366] Some studies have attempted to explore such approaches in the form of systematic reviews [175] or robust animal model characterization [367].

Additionally, with the robust alignment of disease features in preclinical models and human MAFLD, further stratification can be performed to move toward greater disease precision. With robust model characterization and stratification, aligned underlying disease pathogenesis can be more closely recapitulated. This would present a crucial opportunity to represent a monumental shift in studies toward understanding how different metabolic disease subtypes can result in different disease outcomes [368]. Yielding such insights could inform disease management and repurposing of drugs to accelerate therapeutic development.

In current preclinical research, it is extremely important to be clear in aligning the biological implications of the disease. These have been demonstrated in drugs currently in clinical trials. For instance, a promising candidate in Phase 3 trials is Liraglutide. Liraglutide is a glucagon-like peptide-1 (GLP-1) inhibitor, a class of metabolic-modulating anti-diabetic drugs. In clinical trials, it was reported to reduce hepatic steatosis and improve insulin resistance and obesity [369], meeting the primary outcomes of most studies. Consistently, in preclinical models, Liraglutide has been shown to attenuate NAFLD exacerbations and improve these metabolic parameters in mice fed a HFD for generally 10–20 weeks [362]. At these durations, the HFD model exhibits steatosis and limited fibrosis, restricting therapeutic intervention at an early NAFLD-NASH state. Conversely, in 6-week MCD models, the same metabolic improvements were not observed [297], suggesting the misalignment of physiological disease modeling against the underlying mechanisms in humans. As such, paying close attention to the underlying mechanism of the disease is important.

Drugs that have failed, such as selonsertib, an ASK1 inhibitor, did not exhibit any anti-fibrotic effects in patients. However, preclinical MCD and HFCDAA models exhibited improvements in steatosis and fibrosis [368]. For pentoxifylline (PTX), NAFLD models were mostly established with rodents fed an MCD diet. In rodents, it was reported that PTX treatment resulted in a significant reduction in NASH, serum ALT and inflammation. However, treatment resulted in increased hepatic TG. In an RCT, subjects administered PTX did not have improved NASH or hepatic steatosis and discontinued [370].

Other than comprehensive alignment and characterization of preclinical models to human conditions, the liver phenotype must also be taken into account. In the experimental design of interventional studies, the liver phenotype of the MAFLD model at that point of However, in studying metabolic associated liver disease exacerbations, metabolic-associated DIO models require 30–50 weeks to achieve modest F2 liver fibrosis staging, which is not cost and time efficient. As such, there are studies that suggest introducing a combination of modest choline deficiency as a way to exacerbate disease progression [359]. Although conceivable, the utility of such a model requires further characterization of the underlying biology.

Last, as clinical genetic studies continue to expand in MAFLD and its associated metabolic disorders, the development of specific genetic and genetic-DIO models is crucial. The gene variant *PNPLA3* (*rs738409* C > G), encoding the I148M variant, is widely studied in MAFLD. Extensive work has been performed to unravel the underlying loss of glycerolipid hydrolytic function. However, PNPLA3 I148M knock-in mouse experiments enabled novel biological functions to be identified, and the PNPLA3 I148M variant also exhibits a gain-in-function of being resistant to proteasome degradation. As such, this causes an overaccumulation of aberrant PNPLA3-I148M which remodels lipid droplet homeostasis. Consequently, the novel elucidation of the PNPLA-I148M molecular basis allowed for the innovation of RNAi technology as a therapeutic, which is currently in clinical trials (NCT04142424, NCT04483947) [303]. The establishment of double knockout systems and characterization of both genetic and metabolic factors could further provide insights into the drivers of the disease given recent work toward describing a polygenic risk scoring system for MAFLD [371,372].

## Figures and Tables

**Table 1:** Summary of commonly used animal models. Details of the animal models are compiled and reviewed from [171,172,173,174,175,176,177,178,179,180,181,182]. DIO refers to a diet-induced obesogenic diet; x refers to previous reporting of nonexistent features of the model.

		Model Characteristics		Liver Phenotype					Metabolic Disorder Phenotype			
Category	Subcategory	Model	Diet	Steatosis	Hepatic Ballooning	NASH	Fibrosis	HCC	Obesity	Impaired Glucose Tolerance	Dyslipidemia	Extrahepatic Comorbidities
Mouse Models												
Metabolic	DIO	HFD	60% kcal fat	✓ 10 wks	✓ 36 wks	✓ 52 wks	✓ F1 only 50 wks	x	✓	✓ 10–12 wks	✓ 10–12 wks	✓ CKD
Metabolic	DIO	HFC (HFD + cholesterol)	>40% kcal fat, >1% cholesterol	✓ 12 wks	✓ 12 wks	✓	✓ F1 30 wks	✓ 56 wks	✓	x	✓	✓ Atherosclerosis
Metabolic	DIO	HFD + Atherogenic	60% fat, 1.25% cholesterol, 0.5% cholate	✓ 6 wks	✓ 6 wks	✓ 6 wks	✓ F3 24 wks	x	✓	✓ 24 wks	✓ 6 wks	✓ Atherosclerosis
Metabolic	DIO	ALIOS	45% kcal fat (34% (w/w) trans-fat), 20% sucrose, high-fructose corn syrup drinking water (42 g/L).	✓ 8 wks	✓ 8–16 wks	✓ 16–24 wks	✓ F3 52 wks	✓ 52 wks	✓	✓ 6 wks	✓ 8 wks	
Metabolic	DIO	Western Diet (WD)	~42% kcal fat, 0.2–1% cholesterol, high sucrose	✓	✓	✓ depends on diet	✓ F1 52 wks	x	✓	✓	✓	✓ CKD
Metabolic	DIO	AMLN/GAN	4% kcal fat, 22% fructose, 10% sucrose, 2% cholesterol. AMLN: ~28% (w/w) trans-fat; GAN: 46% saturated fatty acids	✓	✓	✓	✓ F1–F2 28 wks, ✓ F3 50 wks	✓ 80 wks	✓	✓	✓	
Metabolic	Genetic	Leptin (ob/ob); LepR (db/db) Deficiency	Normal diet	✓ 12 wks	x	✓ 30 wks	x	x	✓	✓	✓	
Metabolic	DIO-Genetic	HFD + ob/ob or db/db	HFD	✓ 8 wks	✓	✓ 18 wks	✓ F2 30 wks		✓	✓	✓ 2 wks	
Metabolic	DIO-Genetic	KK-Ay	HFD + fructose + atherogenic	n.r.	n.r.	✓ 4 wks	x	x	✓	✓	✓ 4 wks	
Metabolic	DIO-Genetic	AMLN/GAN + ob/ob	AMLN/GAN	✓	✓	✓	✓ F2-F3 16 wks		✓ 3 wks	✓ 16 wks	✓	
Metabolic	DIO-Genetic	HFC + LDR Deficiency (ldr−/−)	HFC	✓ 3 wks	✓	✓ 3 wks		x	✓	✓ 3 wks	✓ 3 wks	✓ Atherosclerosis ~16 wks
Metabolic	DIO-Genetic	HFD + LDR Deficiency (ldr−/−)	HFD	✓	✓	✓ 6 wks	✓ F3 37 wks	x	✓	✓	✓	✓ Atherosclerosis ~16–24 wks
Metabolic	DIO-Strain	MS-NASH (C57Bl/6 x AKR/J)	WD (21% kcal fat, 0.2% cholesterol, 5% fructose, drinking water	✓ 4–8 wks	✓ 16 wks	✓ 16 wks	✓ F1 20 wks	x	✓	✓	✓	
Metabolic	Diet-Strain	Diamond (C57Bl/6 × S129S1/SvImJ)	WD (42% kcal fat, 0.1% cholesterol, 3.1 g/L d-fructose, 18.9 g/L d-glucose	✓	✓ 24 wks	✓ 24 wks	✓ F2 24–36 wks, ✓ F3 52 wks	✓ 52 wks	✓ 16 wks	✓ 16 wks	✓ 16 wks	
FLD-progression	Nutrient depletion	MCD	no methionine and choline, 40% ucrose, 10% kcal fat	✓ 2 wks	x	✓ 6 wks	✓ F1 7 wks, ✓ F2 14 wks, ✓ F3 16 wks	x	x	x (enhanced sensitivity)	x	
FLD-progression	Nutrient depletion	CDAA	choline-deficient, 68.5% carbohydrate, 17.4% proteins, 14% kcal fat	✓	x	✓	✓ F1 20 wks	x	x	x	x	
FLD-progression	Nutrient depletion	CDAA-HFD	CDAA + 60% kcal fat, 0.1% methionine	✓	x	✓ F1 8 wks, ✓ F3 48 wks	✓ F2 24 wks, ✓ F3 48 wks	✓ 52–60 wks	x	x	x	
FLD-progression	Chemical	HFD + CCl4 (simultaneous)	60% kcal fat	✓ (CCl4-dose dependent	✓ (CCl4-dose dependent)	✓ (CCl4-dose dependent)	✓ possible severe fibrosis, CCl4-dose dependent	✓ 24 wks	✓	✓	x	
FLD-progression	Chemical	WD + CCl4 (sequential)	40% kcal fat + 5% fructose	n.r.	n.r.	n.r.	✓ F2–3 2–6 wks, ✓ F4 9 wks	✓ 24 wks	✓	✓	✓	
FLD-progression	Chemical	HFD + DEN	60% kcal fat	n.r.	n.r.	x	x	✓ 20 wks	✓	✓	✓	
Pathogenesis	Genetic	MUP-uPA	HFD	✓	✓	✓ 16 wks	✓ 24 wks	✓ 40 wks	✓	✓	✓	
**Rat Models**												
Metabolic	DIO	HFD	~60% kcal fat	✓ 6–8 wks	x	✓ limited	✓ limited	x	✓ ~7 wks	✓	✓ 7 wks	
Metabolic	DIO	HFD + cholesterol	~65% kcal fat, 1% cholesterol, 0.25% cholate	✓	✓ 16 wks (mild)	✓ limited	✓ F2 16–20 wks	x	✓ ~7 wks	✓ ~4 wks	✓ ~12 wks	
Metabolic	DIO-Genetic	SHR	Chow diet	✓ 8 wks	✓ 16 months	✓ 16 months	x	x	x	✓ ~16 months	✓ 10 wks	
Metabolic	Genetic	OLETF rats	Chow diet	✓ 22–38 wks	x	x	x	x	✓ 8 wks	✓ 18 wks	✓ 18 wks	
Metabolic	DIO-Genetic	OLETF + MCDD	MCDD at 24 wks age	✓ 8 wks	✓ 8 wks	✓ 8 wks	✓ F2 8 wks	x	✓ 8 wks	✓ 8 wks	✓ 8 wks	
Metabolic	DIO-Genetic	Zucker fatty rats (ZFR)	HFD						✓	x	✓	
FLD-progression	Nutrient depletion	Wistar rats + MCD	MCD	✓ 13 wks	x	x	x	x	x (weight loss)	x	x	
FLD-progression	Nutrient depletion	Wistar rats + CDAA	CDAA	✓ 6–12 wks	✓	✓	✓ F3 12 wks	✓ 40 wks (50% of cases)	x	x	x	
**Rabbit Models**												
Metabolic	DIO	HFD + cholesterol	10% lard + 2% cholesterol	✓ 12 wks	✓	✓	✓ F3–F4 36 wks (12% oil + 0.75% cholesterol)	x	x	x	✓	
**Pig Models**												
Metabolic	DIO	Ossabow pigs + High fructose diet	10.5% kcal fat, 20% kcal fructose	x	x	x	x	x	✓ 24 wks	✓ 24 wks	✓ 24 wks	
Metabolic	DIO	Ossabow pigs + HFD + fructose diet	43% kcal fat, 18% kcal fructose	x	✓ 24 wks	x	✓ 24 wks	x	✓ 24 wks	✓ 24 wks	✓ 24 wks	
Metabolic	DIO	Ossabow pigs + HFC + atherogenic	43% kcal fat, 18–20% kcal fructose + 2% cholesterol + reduced cholate	✓ 8 wks	✓16–24 wks	x	✓ F2 16–24 wks	x	✓ 8 wks	x	✓ 8 wks	
Metabolic	DIO-Genetic	LEPTIN−/−	Chow diet	✓ 6–12 months	✓ 12–22 months	✓ 12–22 months, limited	✓ F1–F2 22–35 months	x	✓ 8 wks	x	✓ 12–18 months	

(wks denotes weeks).

## Data Availability

Not applicable.

## References

[1] Araujo A.R., Rosso N., Bedogni G., Tiribelli C., Bellentani S. (2018). Global epidemiology of non-alcoholic fatty liver disease/non-alcoholic steatohepatitis: What we need in the future. Liver Int..

[2] Muthiah M.D., Sanyal A.J. (2020). Burden of Disease due to Nonalcoholic Fatty Liver Disease. Gastroenterol. Clin. N. Am..

[3] Godoy-Matos A.F., Silva Junior W.S., Valerio C.M. (2020). NAFLD as a continuum: From obesity to metabolic syndrome and diabetes. Diabetol. Metab. Syndr..

[4] Lin H., Zhang X., Li G., Wong G.L., Wong V.W. (2021). Epidemiology and Clinical Outcomes of Metabolic (Dysfunction)-associated Fatty Liver Disease. J. Clin. Transl. Hepatol..

[5] Day C.P., James O.F. (1998). Steatohepatitis: A tale of two “hits”?. Gastroenterology.

[6] Leamy A.K., Egnatchik R.A., Young J.D. (2013). Molecular mechanisms and the role of saturated fatty acids in the progression of non-alcoholic fatty liver disease. Prog. Lipid Res..

[7] Tilg H., Moschen A.R. (2010). Evolution of inflammation in nonalcoholic fatty liver disease: The multiple parallel hits hypothesis. Hepatology.

[8] Tilg H., Adolph T., Moschen A. (2020). Multiple parallel hits hypothesis in NAFLD e revisited after a decade. Hepatology.

[9] Yamaguchi K., Yang L., McCall S., Huang J., Yu X.X., Pandey S.K., Bhanot S., Monia B.P., Li Y.X., Diehl A.M. (2007). Inhibiting triglyceride synthesis improves hepatic steatosis but exacerbates liver damage and fibrosis in obese mice with nonalcoholic steatohepatitis. Hepatology.

[10] Bellentani S., Marino M. (2009). Epidemiology and natural history of non-alcoholic fatty liver disease (NAFLD). Ann. Hepatol..

[11] Pagano G., Pacini G., Musso G., Gambino R., Mecca F., Depetris N., Cassader M., David E., Cavallo-Perin P., Rizzetto M. (2002). Nonalcoholic steatohepatitis, insulin resistance, and metabolic syndrome: Further evidence for an etiologic association. Hepatology.

[12] Sanyal A.J., Campbell-Sargent C., Mirshahi F., Rizzo W.B., Contos M.J., Sterling R.K., Luketic V.A., Shiffman M.L., Clore J.N. (2001). Nonalcoholic steatohepatitis: Association of insulin resistance and mitochondrial abnormalities. Gastroenterology.

[13] Cusi K. (2009). Role of insulin resistance and lipotoxicity in non-alcoholic steatohepatitis. Clin. Liver Dis..

[14] Guilherme A., Virbasius J.V., Puri V., Czech M.P. (2008). Adipocyte dysfunctions linking obesity to insulin resistance and type 2 diabetes. Nat. Rev. Mol. Cell Biol..

[15] Kisseleva T., Uchinami H., Feirt N., Quintana-Bustamante O., Segovia J.C., Schwabe R.F., Brenner D.A. (2006). Bone marrow-derived fibrocytes participate in pathogenesis of liver fibrosis. J. Hepatol..

[16] Paradies G., Paradies V., Ruggiero F.M., Petrosillo G. (2014). Oxidative stress, cardiolipin and mitochondrial dysfunction in nonalcoholic fatty liver disease. World J. Gastroenterol..

[17] Crespo J., Cayon A., Fernandez-Gil P., Hernandez-Guerra M., Mayorga M., Dominguez-Diez A., Fernandez-Escalante J.C., Pons-Romero F. (2001). Gene expression of tumor necrosis factor alpha and TNF-receptors, p55 and p75, in nonalcoholic steatohepatitis patients. Hepatology.

[18] Zou J., Lai B., Zheng M., Chen Q., Jiang S., Song A., Huang Z., Shi P., Tu X., Wang D. (2018). CD4+ T cells memorize obesity and promote weight regain. Cell. Mol. Immunol..

[19] Hrncir T., Hrncirova L., Kverka M., Hromadka R., Machova V., Trckova E., Kostovcikova K., Kralickova P., Krejsek J., Tlaskalova-Hogenova H. (2021). Gut Microbiota and NAFLD: Pathogenetic Mechanisms, Microbiota Signatures, and Therapeutic Interventions. Microorganisms.

[20] Safari Z., Gerard P. (2019). The links between the gut microbiome and non-alcoholic fatty liver disease (NAFLD). Cell. Mol. Life Sci..

[21] Turnbaugh P.J., Ley R.E., Mahowald M.A., Magrini V., Mardis E.R., Gordon J.I. (2006). An obesity-associated gut microbiome with increased capacity for energy harvest. Nature.

[22] An L., Wirth U., Koch D., Schirren M., Drefs M., Koliogiannis D., Niess H., Andrassy J., Guba M., Bazhin A.V. (2022). The Role of Gut-Derived Lipopolysaccharides and the Intestinal Barrier in Fatty Liver Diseases. J. Gastrointest. Surg..

[23] Krawczyk M., Rau M., Schattenberg J.M., Bantel H., Pathil A., Demir M., Kluwe J., Boettler T., Lammert F., Geier A. (2017). Combined effects of the PNPLA3 rs738409, TM6SF2 rs58542926, and MBOAT7 rs641738 variants on NAFLD severity: A multicenter biopsy-based study. J. Lipid Res..

[24] Sookoian S., Pirola C.J. (2011). Meta-analysis of the influence of I148M variant of patatin-like phospholipase domain containing 3 gene (PNPLA3) on the susceptibility and histological severity of nonalcoholic fatty liver disease. Hepatology.

[25] Eslam M., Newsome P.N., Sarin S.K., Anstee Q.M., Targher G., Romero-Gomez M., Zelber-Sagi S., Wai-Sun Wong V., Dufour J.F., Schattenberg J.M. (2020). A new definition for metabolic dysfunction-associated fatty liver disease: An international expert consensus statement. J. Hepatol..

[26] Dyson J.K., Anstee Q.M., McPherson S. (2014). Non-alcoholic fatty liver disease: A practical approach to diagnosis and staging. Frontline Gastroenterol..

[27] Imajo K., Kessoku T., Honda Y., Tomeno W., Ogawa Y., Mawatari H., Fujita K., Yoneda M., Taguri M., Hyogo H. (2016). Magnetic Resonance Imaging More Accurately Classifies Steatosis and Fibrosis in Patients With Nonalcoholic Fatty Liver Disease Than Transient Elastography. Gastroenterology.

[28] Targher G., Corey K.E., Byrne C.D., Roden M. (2021). The complex link between NAFLD and type 2 diabetes mellitus—Mechanisms and treatments. Nat. Rev. Gastroenterol. Hepatol..

[29] Lassale C., Tzoulaki I., Moons K.G.M., Sweeting M., Boer J., Johnson L., Huerta J.M., Agnoli C., Freisling H., Weiderpass E. (2018). Separate and combined associations of obesity and metabolic health with coronary heart disease: A pan-European case-cohort analysis. Eur. Heart J..

[30] Ampuero J., Aller R., Gallego-Duran R., Banales J.M., Crespo J., Garcia-Monzon C., Pareja M.J., Vilar-Gomez E., Caballeria J., Escudero-Garcia D. (2018). The effects of metabolic status on non-alcoholic fatty liver disease-related outcomes, beyond the presence of obesity. Aliment. Pharmacol. Ther..

[31] Kumar R., Mohan S. (2017). Non-alcoholic Fatty Liver Disease in Lean Subjects: Characteristics and Implications. J. Clin. Transl. Hepatol..

[32] Fan J.G., Kim S.U., Wong V.W. (2017). New trends on obesity and NAFLD in Asia. J. Hepatol..

[33] Brunt E.M., Kleiner D.E., Wilson L.A., Belt P., Neuschwander-Tetri B.A., Network N.C.R. (2011). Nonalcoholic fatty liver disease (NAFLD) activity score and the histopathologic diagnosis in NAFLD: Distinct clinicopathologic meanings. Hepatology.

[34] Liang Y., Chen H., Liu Y., Hou X., Wei L., Bao Y., Yang C., Zong G., Wu J., Jia W. (2022). Association of MAFLD With Diabetes, Chronic Kidney Disease, and Cardiovascular Disease: A 4.6-Year Cohort Study in China. J. Clin. Endocrinol. Metab..

[35] Lin S., Huang J., Wang M., Kumar R., Liu Y., Liu S., Wu Y., Wang X., Zhu Y. (2020). Comparison of MAFLD and NAFLD diagnostic criteria in real world. Liver Int..

[36] Wolf W.J. (1986). Echocardiographic detection of a left atrial thrombus in an infant with complex congenital heart disease. Am. Heart J..

[37] Lim G.E.H., Tang A., Ng C.H., Chin Y.H., Lim W.H., Tan D.J.H., Yong J.N., Xiao J., Lee C.W., Chan M. (2021). An Observational Data Meta-analysis on the Differences in Prevalence and Risk Factors Between MAFLD vs NAFLD. Clin. Gastroenterol. Hepatol..

[38] Ciardullo S., Perseghin G. (2021). Prevalence of NAFLD, MAFLD and associated advanced fibrosis in the contemporary United States population. Liver Int..

[39] Kim D., Konyn P., Sandhu K.K., Dennis B.B., Cheung A.C., Ahmed A. (2021). Metabolic dysfunction-associated fatty liver disease is associated with increased all-cause mortality in the United States. J. Hepatol..

[40] Ayada I., van Kleef L.A., Alferink L.J.M., Li P., de Knegt R.J., Pan Q. (2022). Systematically comparing epidemiological and clinical features of MAFLD and NAFLD by meta-analysis: Focusing on the non-overlap groups. Liver Int..

[41] Wong V.W., Lazarus J.V. (2021). Prognosis of MAFLD vs. NAFLD and implications for a nomenclature change. J. Hepatol..

[42] Eslam M., George J. (2021). MAFLD: A holistic view to redefining fatty liver disease. J. Hepatol..

[43] Chen X., Chen S., Pang J., Tang Y., Ling W. (2021). Are the different MAFLD subtypes based on the inclusion criteria correlated with all-cause mortality?. J. Hepatol..

[44] Sohn W., Kwon H.J., Chang Y., Ryu S., Cho Y.K. (2022). Liver Fibrosis in Asians With Metabolic Dysfunction-Associated Fatty Liver Disease. Clin. Gastroenterol. Hepatol..

[45] Heyens L.J.M., Busschots D., Koek G.H., Robaeys G., Francque S. (2021). Liver Fibrosis in Non-alcoholic Fatty Liver Disease: From Liver Biopsy to Non-invasive Biomarkers in Diagnosis and Treatment. Front. Med..

[46] van Kleef L.A., Ayada I., Alferink L.J.M., Pan Q., de Knegt R.J. (2022). Metabolic dysfunction-associated fatty liver disease improves detection of high liver stiffness: The Rotterdam Study. Hepatology.

[47] Yamamura S., Eslam M., Kawaguchi T., Tsutsumi T., Nakano D., Yoshinaga S., Takahashi H., Anzai K., George J., Torimura T. (2020). MAFLD identifies patients with significant hepatic fibrosis better than NAFLD. Liver Int..

[48] Wang X., Wu S., Yuan X., Chen S., Fu Q., Sun Y., Lan Y., Hu S., Wang Y., Lu Y. (2022). Metabolic Dysfunction-associated Fatty Liver Disease and Mortality Among Chinese Adults: A Prospective Cohort Study. J. Clin. Endocrinol. Metab..

[49] Niriella M.A., Ediriweera D.S., Kasturiratne A., De Silva S.T., Dassanayaka A.S., De Silva A.P., Kato N., Pathmeswaran A., Wickramasinghe A.R., de Silva H.J. (2021). Outcomes of NAFLD and MAFLD: Results from a community-based, prospective cohort study. PLoS ONE.

[50] Kawaguchi T., Tsutsumi T., Nakano D., Eslam M., George J., Torimura T. (2022). MAFLD enhances clinical practice for liver disease in the Asia-Pacific region. Clin. Mol. Hepatol..

[51] Ciardullo S., Monti T., Perseghin G. (2021). Prevalence of Liver Steatosis and Fibrosis Detected by Transient Elastography in Adolescents in the 2017-2018 National Health and Nutrition Examination Survey. Clin. Gastroenterol. Hepatol..

[52] Wong V.W., Wong G.L., Woo J., Abrigo J.M., Chan C.K., Shu S.S., Leung J.K., Chim A.M., Kong A.P., Lui G.C. (2021). Impact of the New Definition of Metabolic Associated Fatty Liver Disease on the Epidemiology of the Disease. Clin. Gastroenterol. Hepatol..

[53] De A., Ahmad N., Mehta M., Singh P., Duseja A. (2022). NAFLD vs. MAFLD—It is not the name but the disease that decides the outcome in fatty liver. J. Hepatol..

[54] Niederseer D., Wernly B., Aigner E., Stickel F., Datz C. (2021). NAFLD and Cardiovascular Diseases: Epidemiological, Mechanistic and Therapeutic Considerations. J. Clin. Med..

[55] Gutierrez-Cuevas J., Santos A., Armendariz-Borunda J. (2021). Pathophysiological Molecular Mechanisms of Obesity: A Link between MAFLD and NASH with Cardiovascular Diseases. Int. J. Mol. Sci..

[56] Miptah H.N., Ramli A.S., Mohamad M., Hashim H., Tharek Z. (2020). Non-alcoholic fatty liver disease (NAFLD) and the cardiovascular disease (CVD) risk categories in primary care: Is there an association?. BMC Fam. Pract..

[57] Byrne C.D., Targher G. (2015). NAFLD: A multisystem disease. J. Hepatol..

[58] George J., Gish R.G., Geier A. (2021). MAFLD and Cardiovascular Events: What Does the Evidence Show?. Clin. Gastroenterol. Hepatol..

[59] Lee H., Lee Y.H., Kim S.U., Kim H.C. (2021). Metabolic Dysfunction-Associated Fatty Liver Disease and Incident Cardiovascular Disease Risk: A Nationwide Cohort Study. Clin. Gastroenterol. Hepatol..

[60] Quek J., Ng C.H., Tang A.S.P., Chew N., Chan M., Khoo C.M., Wei C.P., Chin Y.H., Tay P., Lim G. (2022). Metabolic Associated Fatty Liver Disease Increases the Risk of Systemic Complications and Mortality. A Meta-Analysis and Systematic Review of 12 620 736 Individuals. Endocr. Pract..

[61] Guo Y., Yang J., Ma R., Zhang X., Guo H., He J., Wang X., Cao B., Maimaitijiang R., Li Y. (2022). Metabolic Dysfunction-Associated Fatty Liver Disease Is Associated with the Risk of Incident Cardiovascular Disease: A Prospective Cohort Study in Xinjiang. Nutrients.

[62] Guerreiro G.T.S., Longo L., Fonseca M.A., de Souza V.E.G., Alvares-da-Silva M.R. (2021). Does the risk of cardiovascular events differ between biopsy-proven NAFLD and MAFLD?. Hepatol. Int..

[63] Matsubayashi Y., Fujihara K., Yamada-Harada M., Mitsuma Y., Sato T., Yaguchi Y., Osawa T., Yamamoto M., Kitazawa M., Yamada T. (2022). Impact of metabolic syndrome and metabolic dysfunction-associated fatty liver disease on cardiovascular risk by the presence or absence of type 2 diabetes and according to sex. Cardiovasc. Diabetol..

[64] Tsutsumi T., Eslam M., Kawaguchi T., Yamamura S., Kawaguchi A., Nakano D., Koseki M., Yoshinaga S., Takahashi H., Anzai K. (2021). MAFLD better predicts the progression of atherosclerotic cardiovascular risk than NAFLD: Generalized estimating equation approach. Hepatol. Res..

[65] Mantovani A., Petracca G., Beatrice G., Csermely A., Lonardo A., Schattenberg J.M., Tilg H., Byrne C.D., Targher G. (2022). Non-alcoholic fatty liver disease and risk of incident chronic kidney disease: An updated meta-analysis. Gut.

[66] Mantovani A., Zaza G., Byrne C.D., Lonardo A., Zoppini G., Bonora E., Targher G. (2018). Nonalcoholic fatty liver disease increases risk of incident chronic kidney disease: A systematic review and meta-analysis. Metabolism.

[67] Musso G., Gambino R., Tabibian J.H., Ekstedt M., Kechagias S., Hamaguchi M., Hultcrantz R., Hagstrom H., Yoon S.K., Charatcharoenwitthaya P. (2014). Association of non-alcoholic fatty liver disease with chronic kidney disease: A systematic review and meta-analysis. PLoS Med..

[68] Sun D.Q., Jin Y., Wang T.Y., Zheng K.I., Rios R.S., Zhang H.Y., Targher G., Byrne C.D., Yuan W.J., Zheng M.H. (2021). MAFLD and risk of CKD. Metabolism.

[69] Wang T.Y., Wang R.F., Bu Z.Y., Targher G., Byrne C.D., Sun D.Q., Zheng M.H. (2022). Association of metabolic dysfunction-associated fatty liver disease with kidney disease. Nat. Rev. Nephrol..

[70] Mantovani A., Lombardi R., Cattazzo F., Zusi C., Cappelli D., Dalbeni A. (2022). MAFLD and CKD: An Updated Narrative Review. Int. J. Mol. Sci..

[71] Meijnikman A.S., Davids M., Herrema H., Aydin O., Tremaroli V., Rios-Morales M., Levels H., Bruin S., de Brauw M., Verheij J. (2022). Microbiome-derived ethanol in nonalcoholic fatty liver disease. Nat. Med..

[72] Wang H., Mehal W., Nagy L.E., Rotman Y. (2021). Immunological mechanisms and therapeutic targets of fatty liver diseases. Cell. Mol. Immunol..

[73] Wondmkun Y.T. (2020). Obesity, insulin resistance, and type 2 diabetes: Associations and therapeutic implications. Diabetes Metab. Syndr. Obes. Targets Ther..

[74] Lim J.U., Lee J.H., Kim J.S., Hwang Y.I., Kim T.H., Lim S.Y., Yoo K.H., Jung K.S., Kim Y.K., Rhee C.K. (2017). Comparison of World Health Organization and Asia-Pacific body mass index classifications in COPD patients. Int. J. Chronic Obstr. Pulm. Dis..

[75] Ross R., Neeland I.J., Yamashita S., Shai I., Seidell J., Magni P., Santos R.D., Arsenault B., Cuevas A., Hu F.B. (2020). Waist circumference as a vital sign in clinical practice: A Consensus Statement from the IAS and ICCR Working Group on Visceral Obesity. Nat. Rev. Endocrinol..

[76] Nguyen-Duy T.B., Nichaman M.Z., Church T.S., Blair S.N., Ross R. (2003). Visceral fat and liver fat are independent predictors of metabolic risk factors in men. Am. J. Physiol. Endocrinol. Metab..

[77] Miyazaki Y., Glass L., Triplitt C., Wajcberg E., Mandarino L.J., DeFronzo R.A. (2002). Abdominal fat distribution and peripheral and hepatic insulin resistance in type 2 diabetes mellitus. Am. J. Physiol. Endocrinol. Metab..

[78] Golabi P., Paik J.M., Arshad T., Younossi Y., Mishra A., Younossi Z.M. (2020). Mortality of NAFLD According to the Body Composition and Presence of Metabolic Abnormalities. Hepatol. Commun..

[79] Wattacheril J., Sanyal A.J. (2016). Lean NAFLD: An underrecognized outlier. Curr. Hepatol. Rep..

[80] Gustafson B., Hedjazifar S., Gogg S., Hammarstedt A., Smith U. (2015). Insulin resistance and impaired adipogenesis. Trends Endocrinol. Metab..

[81] Hanlon C.L., Yuan L. (2022). Nonalcoholic Fatty Liver Disease: The Role of Visceral Adipose Tissue. Clin. Liver Dis..

[82] Hydes T., Alam U., Cuthbertson D.J. (2021). The Impact of Macronutrient Intake on Non-alcoholic Fatty Liver Disease (NAFLD): Too Much Fat, Too Much Carbohydrate, or Just Too Many Calories?. Front. Nutr..

[83] Lambert J.E., Ramos-Roman M.A., Browning J.D., Parks E.J. (2014). Increased de novo lipogenesis is a distinct characteristic of individuals with nonalcoholic fatty liver disease. Gastroenterology.

[84] Nusrianto R., Ayundini G., Kristanti M., Astrella C., Amalina N., Riyadina W., Tahapary D.L., Soewondo P. (2019). Visceral adiposity index and lipid accumulation product as a predictor of type 2 diabetes mellitus: The Bogor cohort study of non-communicable diseases risk factors. Diabetes Res. Clin. Pract..

[85] Haczeyni F., Bell-Anderson K.S., Farrell G. (2018). Causes and mechanisms of adipocyte enlargement and adipose expansion. Obes. Rev..

[86] Liang X., Yang Q., Fu X., Rogers C.J., Wang B., Pan H., Zhu M.J., Nathanielsz P.W., Du M. (2016). Maternal obesity epigenetically alters visceral fat progenitor cell properties in male offspring mice. J. Physiol..

[87] Laforest S., Labrecque J., Michaud A., Cianflone K., Tchernof A. (2015). Adipocyte size as a determinant of metabolic disease and adipose tissue dysfunction. Crit. Rev. Clin. Lab. Sci..

[88] Hsiao W.-Y., Jung S.M., Tang Y., Haley J.A., Li R., Li H., Calejman C.M., Sanchez-Gurmaches J., Hung C.-M., Luciano A.K. (2020). The lipid handling capacity of subcutaneous fat is programmed by mTORC2 during development. Cell Rep..

[89] Straczkowski M., Kowalska I. (2008). The role of skeletal muscle sphingolipids in the development of insulin resistance. Rev. Diabet. Stud..

[90] Boren J., Taskinen M.R., Olofsson S.O., Levin M. (2013). Ectopic lipid storage and insulin resistance: A harmful relationship. J. Intern. Med..

[91] Alves-Bezerra M., Cohen D.E. (2017). Triglyceride Metabolism in the Liver. Compr. Physiol..

[92] Listenberger L.L., Han X., Lewis S.E., Cases S., Farese R.V., Ory D.S., Schaffer J.E. (2003). Triglyceride accumulation protects against fatty acid-induced lipotoxicity. Proc. Natl. Acad. Sci. USA.

[93] Jornayvaz F.R., Birkenfeld A.L., Jurczak M.J., Kanda S., Guigni B.A., Jiang D.C., Zhang D., Lee H.Y., Samuel V.T., Shulman G.I. (2011). Hepatic insulin resistance in mice with hepatic overexpression of diacylglycerol acyltransferase 2. Proc. Natl. Acad. Sci. USA.

[94] Alkhouri N., Dixon L.J., Feldstein A.E. (2009). Lipotoxicity in nonalcoholic fatty liver disease: Not all lipids are created equal. Expert Rev. Gastroenterol. Hepatol..

[95] Mota M., Banini B.A., Cazanave S.C., Sanyal A.J. (2016). Molecular mechanisms of lipotoxicity and glucotoxicity in nonalcoholic fatty liver disease. Metabolism.

[96] Yuan L., Terrrault N.A. (2020). PNPLA3 and nonalcoholic fatty liver disease: Towards personalized medicine for fatty liver. Hepatobiliary Surg. Nutr..

[97] Barbara M., Scott A., Alkhouri N. (2018). New insights into genetic predisposition and novel therapeutic targets for nonalcoholic fatty liver disease. Hepatobiliary Surg. Nutr..

[98] Romeo S., Kozlitina J., Xing C., Pertsemlidis A., Cox D., Pennacchio L.A., Boerwinkle E., Cohen J.C., Hobbs H.H. (2008). Genetic variation in PNPLA3 confers susceptibility to nonalcoholic fatty liver disease. Nat. Genet..

[99] BasuRay S., Wang Y., Smagris E., Cohen J.C., Hobbs H.H. (2019). Accumulation of PNPLA3 on lipid droplets is the basis of associated hepatic steatosis. Proc. Natl. Acad. Sci. USA.

[100] Smagris E., Gilyard S., BasuRay S., Cohen J.C., Hobbs H.H. (2016). Inactivation of Tm6sf2, a Gene Defective in Fatty Liver Disease, Impairs Lipidation but Not Secretion of Very Low Density Lipoproteins. J. Biol. Chem..

[101] Stender S., Kozlitina J., Nordestgaard B.G., Tybjaerg-Hansen A., Hobbs H.H., Cohen J.C. (2017). Adiposity amplifies the genetic risk of fatty liver disease conferred by multiple loci. Nat. Genet..

[102] Sookoian S., Pirola C.J. (2019). Genetics of Nonalcoholic Fatty Liver Disease: From Pathogenesis to Therapeutics. Semin. Liver Dis..

[103] Sookoian S., Pirola C.J. (2017). Genetic predisposition in nonalcoholic fatty liver disease. Clin. Mol. Hepatol..

[104] Jacquemyn J., Cascalho A., Goodchild R.E. (2017). The ins and outs of endoplasmic reticulum-controlled lipid biosynthesis. EMBO Rep..

[105] Zhang C., Chen X., Zhu R.M., Zhang Y., Yu T., Wang H., Zhao H., Zhao M., Ji Y.L., Chen Y.H. (2012). Endoplasmic reticulum stress is involved in hepatic SREBP-1c activation and lipid accumulation in fructose-fed mice. Toxicol. Lett..

[106] Kim Y.R., Lee E.J., Shin K.O., Kim M.H., Pewzner-Jung Y., Lee Y.M., Park J.W., Futerman A.H., Park W.J. (2019). Hepatic triglyceride accumulation via endoplasmic reticulum stress-induced SREBP-1 activation is regulated by ceramide synthases. Exp. Mol. Med..

[107] Bhandary B., Marahatta A., Kim H.R., Chae H.J. (2012). An involvement of oxidative stress in endoplasmic reticulum stress and its associated diseases. Int. J. Mol. Sci..

[108] Henkel A., Green R.M. (2013). The unfolded protein response in fatty liver disease. Semin. Liver Dis..

[109] Prasun P., Ginevic I., Oishi K. (2021). Mitochondrial dysfunction in nonalcoholic fatty liver disease and alcohol related liver disease. Transl. Gastroenterol. Hepatol..

[110] Zorov D.B., Juhaszova M., Sollott S.J. (2014). Mitochondrial reactive oxygen species (ROS) and ROS-induced ROS release. Physiol. Rev..

[111] Begriche K., Massart J., Robin M.A., Bonnet F., Fromenty B. (2013). Mitochondrial adaptations and dysfunctions in nonalcoholic fatty liver disease. Hepatology.

[112] Shum M., Ngo J., Shirihai O.S., Liesa M. (2020). Mitochondrial oxidative function in NAFLD: Friend or foe?. Mol. Metab..

[113] Kim R., Emi M., Tanabe K., Murakami S. (2006). Role of the unfolded protein response in cell death. Apoptosis.

[114] Hu H., Tian M., Ding C., Yu S. (2018). The C/EBP Homologous Protein (CHOP) Transcription Factor Functions in Endoplasmic Reticulum Stress-Induced Apoptosis and Microbial Infection. Front. Immunol..

[115] Henkel A.S., Dewey A.M., Anderson K.A., Olivares S., Green R.M. (2012). Reducing endoplasmic reticulum stress does not improve steatohepatitis in mice fed a methionine- and choline-deficient diet. Am. J. Physiol. Gastrointest. Liver Physiol..

[116] Gao X., Guo S., Zhang S., Liu A., Shi L., Zhang Y. (2018). Matrine attenuates endoplasmic reticulum stress and mitochondrion dysfunction in nonalcoholic fatty liver disease by regulating SERCA pathway. J. Transl. Med..

[117] Soon R.K., Yan J.S., Grenert J.P., Maher J.J. (2010). Stress signaling in the methionine-choline-deficient model of murine fatty liver disease. Gastroenterology.

[118] Toriguchi K., Hatano E., Tanabe K., Takemoto K., Nakamura K., Koyama Y., Seo S., Taura K., Uemoto S. (2014). Attenuation of steatohepatitis, fibrosis, and carcinogenesis in mice fed a methionine-choline deficient diet by CCAAT/enhancer-binding protein homologous protein deficiency. J. Gastroenterol. Hepatol..

[119] Feldstein A.E., Wieckowska A., Lopez A.R., Liu Y.C., Zein N.N., McCullough A.J. (2009). Cytokeratin-18 fragment levels as noninvasive biomarkers for nonalcoholic steatohepatitis: A multicenter validation study. Hepatology.

[120] Tamimi T.I., Elgouhari H.M., Alkhouri N., Yerian L.M., Berk M.P., Lopez R., Schauer P.R., Zein N.N., Feldstein A.E. (2011). An apoptosis panel for nonalcoholic steatohepatitis diagnosis. J. Hepatol..

[121] Lahiri P., Schmidt V., Smole C., Kufferath I., Denk H., Strnad P., Rulicke T., Frohlich L.F., Zatloukal K. (2016). p62/Sequestosome-1 Is Indispensable for Maturation and Stabilization of Mallory-Denk Bodies. PLoS ONE.

[122] Caldwell S., Ikura Y., Dias D., Isomoto K., Yabu A., Moskaluk C., Pramoonjago P., Simmons W., Scruggs H., Rosenbaum N. (2010). Hepatocellular ballooning in NASH. J. Hepatol..

[123] Sookoian S., Castano G.O., Scian R., San Martino J., Pirola C.J. (2016). Heat Shock Protein 27 is down-regulated in Ballooned Hepatocytes of Patients with Nonalcoholic Steatohepatitis (NASH). Sci. Rep..

[124] Hirsova P., Ibrahim S.H., Krishnan A., Verma V.K., Bronk S.F., Werneburg N.W., Charlton M.R., Shah V.H., Malhi H., Gores G.J. (2016). Lipid-Induced Signaling Causes Release of Inflammatory Extracellular Vesicles From Hepatocytes. Gastroenterology.

[125] Wen Y., Lambrecht J., Ju C., Tacke F. (2021). Hepatic macrophages in liver homeostasis and diseases-diversity, plasticity and therapeutic opportunities. Cell. Mol. Immunol..

[126] Dominguez M., Miquel R., Colmenero J., Moreno M., Garcia-Pagan J.C., Bosch J., Arroyo V., Gines P., Caballeria J., Bataller R. (2009). Hepatic expression of CXC chemokines predicts portal hypertension and survival in patients with alcoholic hepatitis. Gastroenterology.

[127] Khoury T., Mari A., Nseir W., Kadah A., Sbeit W., Mahamid M. (2019). Neutrophil-to-lymphocyte ratio is independently associated with inflammatory activity and fibrosis grade in nonalcoholic fatty liver disease. Eur J. Gastroenterol. Hepatol..

[128] Wehr A., Baeck C., Ulmer F., Gassler N., Hittatiya K., Luedde T., Neumann U.P., Trautwein C., Tacke F. (2014). Pharmacological inhibition of the chemokine CXCL16 diminishes liver macrophage infiltration and steatohepatitis in chronic hepatic injury. PLoS ONE.

[129] Bataller R., Brenner D.A. (2005). Liver fibrosis. J. Clin. Investig..

[130] Zisser A., Ipsen D.H., Tveden-Nyborg P. (2021). Hepatic Stellate Cell Activation and Inactivation in NASH-Fibrosis-Roles as Putative Treatment Targets?. Biomedicines.

[131] Le Roy T., Llopis M., Lepage P., Bruneau A., Rabot S., Bevilacqua C., Martin P., Philippe C., Walker F., Bado A. (2013). Intestinal microbiota determines development of non-alcoholic fatty liver disease in mice. Gut.

[132] Bergheim I., Weber S., Vos M., Kramer S., Volynets V., Kaserouni S., McClain C.J., Bischoff S.C. (2008). Antibiotics protect against fructose-induced hepatic lipid accumulation in mice: Role of endotoxin. J. Hepatol..

[133] Yao F., Jia R., Huang H., Yu Y., Mei L., Bai L., Ding Y., Zheng P. (2019). Effect of Lactobacillus paracasei N1115 and fructooligosaccharides in nonalcoholic fatty liver disease. Arch. Med. Sci..

[134] Fialho A., Fialho A., Thota P., McCullough A.J., Shen B. (2016). Small Intestinal Bacterial Overgrowth Is Associated with Non-Alcoholic Fatty Liver Disease. J. Gastrointest. Liver Dis..

[135] Wigg A.J., Roberts-Thomson I.C., Dymock R.B., McCarthy P.J., Grose R.H., Cummins A.G. (2001). The role of small intestinal bacterial overgrowth, intestinal permeability, endotoxaemia, and tumour necrosis factor alpha in the pathogenesis of non-alcoholic steatohepatitis. Gut.

[136] Wang B., Jiang X., Cao M., Ge J., Bao Q., Tang L., Chen Y., Li L. (2016). Altered Fecal Microbiota Correlates with Liver Biochemistry in Nonobese Patients with Non-alcoholic Fatty Liver Disease. Sci. Rep..

[137] Li F., Ye J., Shao C., Zhong B. (2021). Compositional alterations of gut microbiota in nonalcoholic fatty liver disease patients: A systematic review and Meta-analysis. Lipids Health Dis..

[138] Mouzaki M., Comelli E.M., Arendt B.M., Bonengel J., Fung S.K., Fischer S.E., McGilvray I.D., Allard J.P. (2013). Intestinal microbiota in patients with nonalcoholic fatty liver disease. Hepatology.

[139] Cushing E.M., Chi X., Sylvers K.L., Shetty S.K., Potthoff M.J., Davies B.S.J. (2017). Angiopoietin-like 4 directs uptake of dietary fat away from adipose during fasting. Mol. Metab..

[140] Janssen A.W.F., Katiraei S., Bartosinska B., Eberhard D., Willems van Dijk K., Kersten S. (2018). Loss of angiopoietin-like 4 (ANGPTL4) in mice with diet-induced obesity uncouples visceral obesity from glucose intolerance partly via the gut microbiota. Diabetologia.

[141] Yang H., Xiang Y., Robinson K., Wang J., Zhang G., Zhao J., Xiao Y. (2018). Gut Microbiota Is a Major Contributor to Adiposity in Pigs. Front. Microbiol..

[142] Hardie D.G., Pan D.A. (2002). Regulation of fatty acid synthesis and oxidation by the AMP-activated protein kinase. Biochem. Soc. Trans..

[143] Mottillo E.P., Desjardins E.M., Crane J.D., Smith B.K., Green A.E., Ducommun S., Henriksen T.I., Rebalka I.A., Razi A., Sakamoto K. (2016). Lack of Adipocyte AMPK Exacerbates Insulin Resistance and Hepatic Steatosis through Brown and Beige Adipose Tissue Function. Cell Metab..

[144] Pollard A.E., Martins L., Muckett P.J., Khadayate S., Bornot A., Clausen M., Admyre T., Bjursell M., Fiadeiro R., Wilson L. (2019). AMPK activation protects against diet induced obesity through Ucp1-independent thermogenesis in subcutaneous white adipose tissue. Nat. Metab..

[145] Jang H.M., Han S.K., Kim J.K., Oh S.J., Jang H.B., Kim D.H. (2019). Lactobacillus sakei Alleviates High-Fat-Diet-Induced Obesity and Anxiety in Mice by Inducing AMPK Activation and SIRT1 Expression and Inhibiting Gut Microbiota-Mediated NF-kappaB Activation. Mol. Nutr. Food Res..

[146] Ding X., Jian T., Li J., Lv H., Tong B., Li J., Meng X., Ren B., Chen J. (2020). Chicoric Acid Ameliorates Nonalcoholic Fatty Liver Disease via the AMPK/Nrf2/NFkappaB Signaling Pathway and Restores Gut Microbiota in High-Fat-Diet-Fed Mice. Oxidative Med. Cell. Longev..

[147] Yoshida H., Ishii M., Akagawa M. (2019). Propionate suppresses hepatic gluconeogenesis via GPR43/AMPK signaling pathway. Arch. Biochem. Biophys..

[148] Wu W., Wang S., Liu Q., Shan T., Wang Y. (2018). Metformin Protects against LPS-Induced Intestinal Barrier Dysfunction by Activating AMPK Pathway. Mol. Pharm..

[149] Gassler N. (2017). Paneth cells in intestinal physiology and pathophysiology. World J. Gastrointest. Pathophysiol..

[150] Rohr M.W., Narasimhulu C.A., Rudeski-Rohr T.A., Parthasarathy S. (2020). Negative Effects of a High-Fat Diet on Intestinal Permeability: A Review. Adv. Nutr..

[151] Sellmann C., Priebs J., Landmann M., Degen C., Engstler A.J., Jin C.J., Garttner S., Spruss A., Huber O., Bergheim I. (2015). Diets rich in fructose, fat or fructose and fat alter intestinal barrier function and lead to the development of nonalcoholic fatty liver disease over time. J. Nutr. Biochem..

[152] Nicoletti A., Ponziani F.R., Biolato M., Valenza V., Marrone G., Sganga G., Gasbarrini A., Miele L., Grieco A. (2019). Intestinal permeability in the pathogenesis of liver damage: From non-alcoholic fatty liver disease to liver transplantation. World J. Gastroenterol..

[153] Zhu L., Baker S.S., Gill C., Liu W., Alkhouri R., Baker R.D., Gill S.R. (2013). Characterization of gut microbiomes in nonalcoholic steatohepatitis (NASH) patients: A connection between endogenous alcohol and NASH. Hepatology.

[154] Liver E.A.F.T.S.O.T. (2018). EASL clinical practice guidelines: Management of hepatocellular carcinoma. J. Hepatol..

[155] Sun F.-R., Wang B.-Y. (2021). Alcohol and Metabolic-associated Fatty Liver Disease. J. Clin. Transl. Hepatol..

[156] Buyco D.G., Martin J., Jeon S., Hooks R., Lin C., Carr R. (2021). Experimental models of metabolic and alcoholic fatty liver disease. World J. Gastroenterol..

[157] Baraona E., Lieber C.S. (1979). Effects of ethanol on lipid metabolism. J. Lipid Res..

[158] Israelsen M., Kim M., Suvitaival T., Madsen B.S., Hansen C.D., Torp N., Trost K., Thiele M., Hansen T., Legido-Quigley C. (2021). Comprehensive lipidomics reveals phenotypic differences in hepatic lipid turnover in ALD and NAFLD during alcohol intoxication. JHEP Rep..

[159] Jafri S.M., Gordon S.C. (2018). Epidemiology of hepatitis C. Clin. Liver Dis..

[160] Soza A., Riquelme A., Arrese M. (2010). Routes of transmission of hepatitis C virus. Ann. Hepatol..

[161] Gupta E., Bajpai M., Choudhary A. (2014). Hepatitis C virus: Screening, diagnosis, and interpretation of laboratory assays. Asian J. Transfus. Sci..

[162] Chaudhari R., Fouda S., Sainu A., Pappachan J.M. (2021). Metabolic complications of hepatitis C virus infection. World J. Gastroenterol..

[163] Patel A., Harrison S.A. (2012). Hepatitis C virus infection and nonalcoholic steatohepatitis. Gastroenterol. Hepatol..

[164] Zeisel S.H., Da Costa K.-A. (2009). Choline: An essential nutrient for public health. Nutr. Rev..

[165] Soret P.A., Magusto J., Housset C., Gautheron J. (2020). In Vitro and In Vivo Models of Non-Alcoholic Fatty Liver Disease: A Critical Appraisal. J. Clin. Med..

[166] Corbin K.D., Zeisel S.H. (2012). Choline metabolism provides novel insights into nonalcoholic fatty liver disease and its progression. Curr. Opin. Gastroenterol..

[167] Liu Y., Zang X., Feng K., Liu S., Zhang J., Lv Z., Xin Y., Yu M. (2022). Lipidomic Determination of Serum Lipids by Ultra-High Performance Liquid Chromatography–Mass Spectrometry (UPLC-MS) for the Characterization of Nonalcoholic Fatty Liver Disease (NAFLD). Anal. Lett..

[168] Tokoro M., Gotoh K., Kudo Y., Hirashita Y., Iwao M., Arakawa M., Endo M., Oribe J., Masaki T., Honda K. (2021). α-Tocopherol suppresses hepatic steatosis by increasing CPT-1 expression in a mouse model of diet-induced nonalcoholic fatty liver disease. Obes. Sci. Pract..

[169] Jahn D., Kircher S., Hermanns H.M., Geier A. (2019). Animal models of NAFLD from a hepatologist’s point of view. Biochim. Biophys. Acta Mol. Basis Dis..

[170] Feuerstadt P., Aroniadis O.C., Svedlund F.L., Garcia M., Stong L., Boules M., Khanna S. (2022). Heterogeneity of Randomized Controlled Trials of Fecal Microbiota Transplantation in Recurrent Clostridioides difficile Infection. Dig. Dis. Sci..

[171] Oligschlaeger Y., Shiri-Sverdlov R. (2020). NAFLD Preclinical Models: More than a Handful, Less of a Concern?. Biomedicines.

[172] Zhong F., Zhou X., Xu J., Gao L. (2020). Rodent models of nonalcoholic fatty liver disease. Digestion.

[173] Febbraio M.A., Reibe S., Shalapour S., Ooi G.J., Watt M.J., Karin M. (2019). Preclinical Models for Studying NASH-Driven HCC: How Useful Are They?. Cell Metab..

[174] Nahon P., Allaire M., Nault J.-C., Paradis V. (2020). Characterizing the mechanism behind the progression of NAFLD to hepatocellular carcinoma. Hepatic Oncol..

[175] Im Y.R., Hunter H., de Gracia Hahn D., Duret A., Cheah Q., Dong J., Fairey M., Hjalmarsson C., Li A., Lim H.K. (2021). A Systematic Review of Animal Models of NAFLD Finds High-Fat, High-Fructose Diets Most Closely Resemble Human NAFLD. Hepatology.

[176] Pouwer M.G., Heinonen S.E., Behrendt M., Andréasson A.-C., van Koppen A., Menke A.L., Pieterman E.J., van den Hoek A.M., Jukema J.W., Leighton B. (2019). The APOE3-leiden heterozygous glucokinase knockout mouse as novel translational disease model for type 2 diabetes, dyslipidemia, and diabetic atherosclerosis. J. Diabetes Res..

[177] Deji N., Kume S., Araki S.-I., Soumura M., Sugimoto T., Isshiki K., Chin-Kanasaki M., Sakaguchi M., Koya D., Haneda M. (2009). Structural and functional changes in the kidneys of high-fat diet-induced obese mice. Am. J. Physiol.-Ren. Physiol..

[178] Wicks S.E., Nguyen T.-T., Breaux C., Kruger C., Stadler K. (2016). Diet-induced obesity and kidney disease—In search of a susceptible mouse model. Biochimie.

[179] Zhang X., Coker O.O., Chu E.S., Fu K., Lau H.C., Wang Y.-X., Chan A.W., Wei H., Yang X., Sung J.J. (2021). Dietary cholesterol drives fatty liver-associated liver cancer by modulating gut microbiota and metabolites. Gut.

[180] Ipsen D.H., Lykkesfeldt J., Tveden-Nyborg P. (2020). Animal Models of Fibrosis in Nonalcoholic Steatohepatitis: Do They Reflect Human Disease?. Adv. Nutr..

[181] Nakagawa H. (2015). Recent advances in mouse models of obesity- and nonalcoholic steatohepatitis-associated hepatocarcinogenesis. World J. Hepatol..

[182] Carreres L., Jílková Z.M., Vial G., Marche P.N., Decaens T., Lerat H. (2021). Modeling Diet-Induced NAFLD and NASH in Rats: A Comprehensive Review. Biomedicines.

[183] Hansen H.H., HM A.E., Oro D., Evers S.S., Heeboll S., Eriksen P.L., Thomsen K.L., Bengtsson A., Veidal S.S., Feigh M. (2020). Human translatability of the GAN diet-induced obese mouse model of non-alcoholic steatohepatitis. BMC Gastroenterol..

[184] Boland M.L., Oro D., Tolbol K.S., Thrane S.T., Nielsen J.C., Cohen T.S., Tabor D.E., Fernandes F., Tovchigrechko A., Veidal S.S. (2019). Towards a standard diet-induced and biopsy-confirmed mouse model of non-alcoholic steatohepatitis: Impact of dietary fat source. World J. Gastroenterol..

[185] Radhakrishnan S., Yeung S.F., Ke J.Y., Antunes M.M., Pellizzon M.A. (2021). Considerations When Choosing High-Fat, High-Fructose, and High-Cholesterol Diets to Induce Experimental Nonalcoholic Fatty Liver Disease in Laboratory Animal Models. Curr. Dev. Nutr..

[186] Eng J.M., Estall J.L. (2021). Diet-Induced Models of Non-Alcoholic Fatty Liver Disease: Food for Thought on Sugar, Fat, and Cholesterol. Cells.

[187] Drescher H.K., Weiskirchen R., Fulop A., Hopf C., de San Roman E.G., Huesgen P.F., de Bruin A., Bongiovanni L., Christ A., Tolba R. (2019). The Influence of Different Fat Sources on Steatohepatitis and Fibrosis Development in the Western Diet Mouse Model of Non-alcoholic Steatohepatitis (NASH). Front. Physiol..

[188] Perakakis N., Stefanakis K., Feigh M., Veidal S.S., Mantzoros C.S. (2021). Elafibranor and liraglutide improve differentially liver health and metabolism in a mouse model of non-alcoholic steatohepatitis. Liver Int..

[189] Gosis B.S., Wada S., Thorsheim C., Li K., Jung S., Rhoades J.H., Yang Y., Brandimarto J., Li L., Uehara K. (2022). Inhibition of nonalcoholic fatty liver disease in mice by selective inhibition of mTORC1. Science.

[190] Mollerhoj M.B., Veidal S.S., Thrane K.T., Oro D., Overgaard A., Salinas C.G., Madsen M.R., Pfisterer L., Vyberg M., Simon E. (2022). Hepatoprotective effects of semaglutide, lanifibranor and dietary intervention in the GAN diet-induced obese and biopsy-confirmed mouse model of NASH. Clin. Transl. Sci..

[191] Heitel P., Faudone G., Helmstädter M., Schmidt J., Kaiser A., Tjaden A., Schröder M., Müller S., Schierle S., Pollinger J. (2020). A triple farnesoid X receptor and peroxisome proliferator-activated receptor α/δ activator reverses hepatic fibrosis in diet-induced NASH in mice. Commun. Chem..

[192] Hosseini Z., Whiting S.J., Vatanparast H. (2016). Current evidence on the association of the metabolic syndrome and dietary patterns in a global perspective. Nutr. Res. Rev..

[193] Yki-Jarvinen H., Luukkonen P.K., Hodson L., Moore J.B. (2021). Dietary carbohydrates and fats in nonalcoholic fatty liver disease. Nat. Rev. Gastroenterol. Hepatol..

[194] de Wit N., Derrien M., Bosch-Vermeulen H., Oosterink E., Keshtkar S., Duval C., de Vogel-van den Bosch J., Kleerebezem M., Muller M., van der Meer R. (2012). Saturated fat stimulates obesity and hepatic steatosis and affects gut microbiota composition by an enhanced overflow of dietary fat to the distal intestine. Am. J. Physiol. Gastrointest. Liver Physiol..

[195] Wang D., Wei Y., Pagliassotti M.J. (2006). Saturated fatty acids promote endoplasmic reticulum stress and liver injury in rats with hepatic steatosis. Endocrinology.

[196] Meex R.C.R., Blaak E.E. (2021). Mitochondrial Dysfunction is a Key Pathway that Links Saturated Fat Intake to the Development and Progression of NAFLD. Mol. Nutr. Food Res..

[197] Cui J., Li L., Ren L., Sun J., Zhao H., Sun Y. (2021). Dietary n-3 and n-6 fatty acid intakes and NAFLD: A cross-sectional study in the United States. Asia Pac. J. Clin. Nutr..

[198] Ducheix S., Piccinin E., Peres C., Garcia-Irigoyen O., Bertrand-Michel J., Fouache A., Cariello M., Lobaccaro J.M., Guillou H., Sabba C. (2022). Reduction in gut-derived MUFAs via intestinal stearoyl-CoA desaturase 1 deletion drives susceptibility to NAFLD and hepatocarcinoma. Hepatol. Commun..

[199] Duwaerts C.C., Amin A.M., Siao K., Her C., Fitch M., Beysen C., Turner S.M., Goodsell A., Baron J.L., Grenert J.P. (2017). Specific Macronutrients Exert Unique Influences on the Adipose-Liver Axis to Promote Hepatic Steatosis in Mice. Cell. Mol. Gastroenterol. Hepatol..

[200] Xie X., Guo B., Xiao X., Yin J., Wang Z., Jiang X., Li J., Long L., Zhou J., Zhang N. (2022). Healthy dietary patterns and metabolic dysfunction-associated fatty liver disease in less-developed ethnic minority regions: A large cross-sectional study. BMC Public Health.

[201] Jung S., Bae H., Song W.S., Jang C. (2022). Dietary Fructose and Fructose-Induced Pathologies. Annu. Rev. Nutr..

[202] Malik V.S., Hu F.B. (2022). The role of sugar-sweetened beverages in the global epidemics of obesity and chronic diseases. Nat. Rev. Endocrinol..

[203] Jensen T., Abdelmalek M.F., Sullivan S., Nadeau K.J., Green M., Roncal C., Nakagawa T., Kuwabara M., Sato Y., Kang D.H. (2018). Fructose and sugar: A major mediator of non-alcoholic fatty liver disease. J. Hepatol..

[204] Helsley R.N., Moreau F., Gupta M.K., Radulescu A., DeBosch B., Softic S. (2020). Tissue-Specific Fructose Metabolism in Obesity and Diabetes. Curr. Diabetes Rep..

[205] Drozdz K., Nabrdalik K., Hajzler W., Kwiendacz H., Gumprecht J., Lip G.Y.H. (2021). Metabolic-Associated Fatty Liver Disease (MAFLD), Diabetes, and Cardiovascular Disease: Associations with Fructose Metabolism and Gut Microbiota. Nutrients.

[206] Wang Y.L., Zhou X., Li D.L., Ye J.M. (2022). Role of the mTOR-autophagy-ER stress pathway in high fructose-induced metabolic-associated fatty liver disease. Acta Pharmacol. Sin..

[207] Liao Y., Davies N.A., Bogle I.D.L. (2020). Computational Modeling of Fructose Metabolism and Development in NAFLD. Front Bioeng Biotechnol..

[208] Vasques-Monteiro I.M.L., Silva-Veiga F.M., Miranda C.S., de Andrade Goncalves E.C.B., Daleprane J.B., Souza-Mello V. (2021). A rise in Proteobacteria is an indicator of gut-liver axis-mediated nonalcoholic fatty liver disease in high-fructose-fed adult mice. Nutr. Res..

[209] Li H., Yu X.H., Ou X., Ouyang X.P., Tang C.K. (2021). Hepatic cholesterol transport and its role in non-alcoholic fatty liver disease and atherosclerosis. Prog. Lipid Res..

[210] Liao X., Ma Q., Wu T., Shao C., Lin Y., Sun Y., Feng S., Wang W., Ye J., Zhong B. (2022). Lipid-Lowering Responses to Dyslipidemia Determine the Efficacy on Liver Enzymes in Metabolic Dysfunction-Associated Fatty Liver Disease with Hepatic Injuries: A Prospective Cohort Study. Diabetes Metab. Syndr. Obes..

[211] Ciardullo S., Perseghin G. (2021). Statin use is associated with lower prevalence of advanced liver fibrosis in patients with type 2 diabetes. Metabolism.

[212] Dongiovanni P., Petta S., Mannisto V., Mancina R.M., Pipitone R., Karja V., Maggioni M., Kakela P., Wiklund O., Mozzi E. (2015). Statin use and non-alcoholic steatohepatitis in at risk individuals. J. Hepatol..

[213] Radhakrishnan S., Ke J.Y., Pellizzon M.A. (2020). Targeted Nutrient Modifications in Purified Diets Differentially Affect Nonalcoholic Fatty Liver Disease and Metabolic Disease Development in Rodent Models. Curr. Dev. Nutr..

[214] Zhang G., Wang X., Chung T.-Y., Ye W., Hodge L., Zhang L., Chng K., Xiao Y.-F., Wang Y.J. (2020). Carbon tetrachloride (CCl4) accelerated development of non-alcoholic fatty liver disease (NAFLD)/steatohepatitis (NASH) in MS-NASH mice fed western diet supplemented with fructose (WDF). BMC Gastroenterol..

[215] Peterson R.G., Jackson C.V., Zimmerman K.M., Alsina-Fernandez J., Michael M.D., Emmerson P.J., Coskun T. (2017). Glucose dysregulation and response to common anti-diabetic agents in the FATZO/Pco mouse. PLoS ONE.

[216] Asgharpour A., Cazanave S.C., Pacana T., Seneshaw M., Vincent R., Banini B.A., Kumar D.P., Daita K., Min H.K., Mirshahi F. (2016). A diet-induced animal model of non-alcoholic fatty liver disease and hepatocellular cancer. J. Hepatol..

[217] Blais E.M., Rawls K.D., Dougherty B.V., Li Z.I., Kolling G.L., Ye P., Wallqvist A., Papin J.A. (2017). Reconciled rat and human metabolic networks for comparative toxicogenomics and biomarker predictions. Nat. Commun..

[218] Zou Y., Li J., Lu C., Wang J., Ge J., Huang Y., Zhang L., Wang Y. (2006). High-fat emulsion-induced rat model of nonalcoholic steatohepatitis. Life Sci..

[219] Ito M., Suzuki J., Tsujioka S., Sasaki M., Gomori A., Shirakura T., Hirose H., Ito M., Ishihara A., Iwaasa H. (2007). Longitudinal analysis of murine steatohepatitis model induced by chronic exposure to high-fat diet. Hepatol. Res..

[220] Charlton M., Krishnan A., Viker K., Sanderson S., Cazanave S., McConico A., Masuoko H., Gores G. (2011). Fast food diet mouse: Novel small animal model of NASH with ballooning, progressive fibrosis, and high physiological fidelity to the human condition. Am. J. Physiol. Gastrointest. Liver Physiol..

[221] Kucera O., Garnol T., Lotkova H., Stankova P., Mazurova Y., Hroch M., Bolehovska R., Rousar T., Cervinkova Z. (2011). The effect of rat strain, diet composition and feeding period on the development of a nutritional model of non-alcoholic fatty liver disease in rats. Physiol. Res..

[222] Ahmed U., Redgrave T.G., Oates P.S. (2009). Effect of dietary fat to produce non-alcoholic fatty liver in the rat. J. Gastroenterol. Hepatol..

[223] Wang Y., Ausman L.M., Russell R.M., Greenberg A.S., Wang X.D. (2008). Increased apoptosis in high-fat diet-induced nonalcoholic steatohepatitis in rats is associated with c-Jun NH2-terminal kinase activation and elevated proapoptotic Bax. J. Nutr..

[224] Buettner R., Ascher M., Gabele E., Hellerbrand C., Kob R., Bertsch T., Bollheimer L.C. (2013). Olive oil attenuates the cholesterol-induced development of nonalcoholic steatohepatitis despite increased insulin resistance in a rodent model. Horm. Metab. Res..

[225] Ichimura M., Kawase M., Masuzumi M., Sakaki M., Nagata Y., Tanaka K., Suruga K., Tamaru S., Kato S., Tsuneyama K. (2015). High-fat and high-cholesterol diet rapidly induces non-alcoholic steatohepatitis with advanced fibrosis in Sprague-Dawley rats. Hepatol. Res..

[226] Haldrup D., Heeboll S., Thomsen K.L., Andersen K.J., Meier M., Mortensen F.V., Nyengaard J.R., Hamilton-Dutoit S., Gronbaek H. (2018). Preserved liver regeneration capacity after partial hepatectomy in rats with non-alcoholic steatohepatitis. World J. Hepatol..

[227] Cai C.X., Buddha H., Castelino-Prabhu S., Zhang Z., Britton R.S., Bacon B.R., Neuschwander-Tetri B.A. (2017). Activation of Insulin-PI3K/Akt-p70S6K Pathway in Hepatic Stellate Cells Contributes to Fibrosis in Nonalcoholic Steatohepatitis. Dig. Dis. Sci..

[228] Xu Z.J., Fan J.G., Ding X.D., Qiao L., Wang G.L. (2010). Characterization of high-fat, diet-induced, non-alcoholic steatohepatitis with fibrosis in rats. Dig. Dis. Sci..

[229] Abdelmalek M.F., Suzuki A., Guy C., Unalp-Arida A., Colvin R., Johnson R.J., Diehl A.M., Nonalcoholic Steatohepatitis Clinical Research N. (2010). Increased fructose consumption is associated with fibrosis severity in patients with nonalcoholic fatty liver disease. Hepatology.

[230] Henkel J., Buchheim-Dieckow K., Castro J.P., Laeger T., Wardelmann K., Kleinridders A., Johrens K., Puschel G.P. (2019). Reduced Oxidative Stress and Enhanced FGF21 Formation in Livers of Endurance-Exercised Rats with Diet-Induced NASH. Nutrients.

[231] Yin Y., Liu H., Zheng Z., Lu R., Jiang Z. (2019). Genistein can ameliorate hepatic inflammatory reaction in nonalcoholic steatohepatitis rats. Biomed. Pharmacother..

[232] Tsuchiya T., Naitoh T., Nagao M., Tanaka N., Watanabe K., Imoto H., Miyachi T., Motoi F., Unno M. (2018). Increased Bile Acid Signals After Duodenal-Jejunal Bypass Improve Non-alcoholic Steatohepatitis (NASH) in a Rodent Model of Diet-Induced NASH. Obes. Surg..

[233] Garcia-Lezana T., Raurell I., Bravo M., Torres-Arauz M., Salcedo M.T., Santiago A., Schoenenberger A., Manichanh C., Genesca J., Martell M. (2018). Restoration of a healthy intestinal microbiota normalizes portal hypertension in a rat model of nonalcoholic steatohepatitis. Hepatology.

[234] Fan J., Kitajima S., Watanabe T., Xu J., Zhang J., Liu E., Chen Y.E. (2015). Rabbit models for the study of human atherosclerosis: From pathophysiological mechanisms to translational medicine. Pharmacol. Ther..

[235] Fan J., Watanabe T. (2003). Transgenic rabbits as therapeutic protein bioreactors and human disease models. Pharmacol. Ther..

[236] Fu J.F., Fang Y.L., Liang L., Wang C.L., Hong F., Dong G.P. (2009). A rabbit model of pediatric nonalcoholic steatohepatitis: The role of adiponectin. World J. Gastroenterol..

[237] Wang Y., Zhang P., Su X., Yu Q., Chen Y., Guan H., Liu E., Fan J. (2018). Establishment of a novel nonalcoholic fatty liver disease model using cholesterolfed rabbits with reference to the potential role of endoplasmic reticulum stress. Mol. Med. Rep..

[238] Ogawa T., Fujii H., Yoshizato K., Kawada N. (2010). A human-type nonalcoholic steatohepatitis model with advanced fibrosis in rabbits. Am. J. Pathol..

[239] Schmidt N.H., Svendsen P., Albarran-Juarez J., Moestrup S.K., Bentzon J.F. (2021). High-fructose feeding does not induce steatosis or non-alcoholic fatty liver disease in pigs. Sci. Rep..

[240] Lee L., Alloosh M., Saxena R., Van Alstine W., Watkins B.A., Klaunig J.E., Sturek M., Chalasani N. (2009). Nutritional model of steatohepatitis and metabolic syndrome in the Ossabaw miniature swine. Hepatology.

[241] Schumacher-Petersen C., Christoffersen B.O., Kirk R.K., Ludvigsen T.P., Zois N.E., Pedersen H.D., Vyberg M., Olsen L.H. (2019). Experimental non-alcoholic steatohepatitis in Gottingen Minipigs: Consequences of high fat-fructose-cholesterol diet and diabetes. J. Transl. Med..

[242] Lim J.S., Mietus-Snyder M., Valente A., Schwarz J.M., Lustig R.H. (2010). The role of fructose in the pathogenesis of NAFLD and the metabolic syndrome. Nat. Rev. Gastroenterol. Hepatol..

[243] Liang T., Alloosh M., Bell L.N., Fullenkamp A., Saxena R., Van Alstine W., Bybee P., Werling K., Sturek M., Chalasani N. (2015). Liver injury and fibrosis induced by dietary challenge in the Ossabaw miniature Swine. PLoS ONE.

[244] Horn C.L., Morales A.L., Savard C., Farrell G.C., Ioannou G.N. (2022). Role of Cholesterol-Associated Steatohepatitis in the Development of NASH. Hepatol. Commun..

[245] HM A.E., Veidal S.S., Feigh M., Hallenborg P., Puglia M., Pers T.H., Vrang N., Jelsing J., Kornum B.R., Blagoev B. (2020). Multi-omics characterization of a diet-induced obese model of non-alcoholic steatohepatitis. Sci. Rep..

[246] Savard C., Tartaglione E.V., Kuver R., Haigh W.G., Farrell G.C., Subramanian S., Chait A., Yeh M.M., Quinn L.S., Ioannou G.N. (2013). Synergistic interaction of dietary cholesterol and dietary fat in inducing experimental steatohepatitis. Hepatology.

[247] Suriano F., Vieira-Silva S., Falony G., Roumain M., Paquot A., Pelicaen R., Regnier M., Delzenne N.M., Raes J., Muccioli G.G. (2021). Novel insights into the genetically obese (ob/ob) and diabetic (db/db) mice: Two sides of the same coin. Microbiome.

[248] Ferguson D., Blenden M., Hutson I., Du Y., Harris C.A. (2018). Mouse Embryonic Fibroblasts Protect ob/ob Mice From Obesity and Metabolic Complications. Endocrinology.

[249] Hudkins K.L., Pichaiwong W., Wietecha T., Kowalewska J., Banas M.C., Spencer M.W., Muhlfeld A., Koelling M., Pippin J.W., Shankland S.J. (2010). BTBR Ob/Ob mutant mice model progressive diabetic nephropathy. J. Am. Soc. Nephrol..

[250] Palmer N.D., Kahali B., Kuppa A., Chen Y., Du X., Feitosa M.F., Bielak L.F., O’Connell J.R., Musani S.K., Guo X. (2021). Allele-specific variation at APOE increases nonalcoholic fatty liver disease and obesity but decreases risk of Alzheimer’s disease and myocardial infarction. Hum. Mol. Genet..

[251] Liu S., Weng R., Gu X., Li L., Zhong Z. (2021). Association between apolipoprotein E gene polymorphism and nonalcoholic fatty liver disease in Southern China: A case-control study. J. Clin. Lab. Anal..

[252] Zheng F., Cai Y. (2019). Concurrent exercise improves insulin resistance and nonalcoholic fatty liver disease by upregulating PPAR-gamma and genes involved in the beta-oxidation of fatty acids in ApoE-KO mice fed a high-fat diet. Lipids Health Dis..

[253] Rinne P., Kadiri J.J., Velasco-Delgado M., Nuutinen S., Viitala M., Hollmen M., Rami M., Savontaus E., Steffens S. (2018). Melanocortin 1 Receptor Deficiency Promotes Atherosclerosis in Apolipoprotein E(−/−) Mice. Arterioscler. Thromb. Vasc. Biol..

[254] Spanos C., Maldonado E.M., Fisher C.P., Leenutaphong P., Oviedo-Orta E., Windridge D., Salguero F.J., Bermudez-Fajardo A., Weeks M.E., Evans C. (2018). Proteomic identification and characterization of hepatic glyoxalase 1 dysregulation in non-alcoholic fatty liver disease. Proteome Sci..

[255] Chen W., Zhang X., Xu M., Jiang L., Zhou M., Liu W., Chen Z., Wang Y., Zou Q., Wang L. (2021). Betaine prevented high-fat diet-induced NAFLD by regulating the FGF10/AMPK signaling pathway in ApoE(−/−) mice. Eur. J. Nutr..

[256] Feng D., Zou J., Su D., Mai H., Zhang S., Li P., Zheng X. (2019). Curcumin prevents high-fat diet-induced hepatic steatosis in ApoE(−/−) mice by improving intestinal barrier function and reducing endotoxin and liver TLR4/NF-kappaB inflammation. Nutr. Metab..

[257] Joven J., Rull A., Ferre N., Escola-Gil J.C., Marsillach J., Coll B., Alonso-Villaverde C., Aragones G., Claria J., Camps J. (2007). The results in rodent models of atherosclerosis are not interchangeable: The influence of diet and strain. Atherosclerosis.

[258] van den Hoek A.M., Verschuren L., Worms N., van Nieuwkoop A., de Ruiter C., Attema J., Menke A.L., Caspers M.P.M., Radhakrishnan S., Salic K. (2020). A Translational Mouse Model for NASH with Advanced Fibrosis and Atherosclerosis Expressing Key Pathways of Human Pathology. Cells.

[259] Zhao Y., Yang Y., Xing R., Cui X., Xiao Y., Xie L., You P., Wang T., Zeng L., Peng W. (2018). Hyperlipidemia induces typical atherosclerosis development in Ldlr and Apoe deficient rats. Atherosclerosis.

[260] Kampschulte M., Stockl C., Langheinrich A.C., Althohn U., Bohle R.M., Krombach G.A., Stieger P., Churin Y., Kremer S., Dierkes C. (2014). Western diet in ApoE-LDLR double-deficient mouse model of atherosclerosis leads to hepatic steatosis, fibrosis, and tumorigenesis. Lab. Investig..

[261] Takahashi Y., Fukusato T., Conn P.M. (2017). Chapter 13—Animal Models of Liver Diseases. Animal Models for the Study of Human Disease.

[262] Sakuma T., Nakamura M., Chiba T., Iwanaga T., Kan M., Kojima R., Ao J., Ma Y., Unozawa H., Fujita N. (2022). A diet-induced murine model for non-alcoholic fatty liver disease with obesity and insulin resistance that rapidly develops steatohepatitis and fibrosis. Lab. Investig..

[263] Liu J.X., Liu J., Li P.Q., Xie X.D., Guo Q., Tian L.M., Ma X.Q., Zhang J.P., Liu J., Gao J.Y. (2008). Association of sterol regulatory element-binding protein-1c gene polymorphism with type 2 diabetes mellitus, insulin resistance and blood lipid levels in Chinese population. Diabetes Res. Clin. Pract..

[264] Eberle D., Clement K., Meyre D., Sahbatou M., Vaxillaire M., Le Gall A., Ferre P., Basdevant A., Froguel P., Foufelle F. (2004). SREBF-1 gene polymorphisms are associated with obesity and type 2 diabetes in French obese and diabetic cohorts. Diabetes.

[265] Qi N.R., Wang J., Zidek V., Landa V., Mlejnek P., Kazdová L., Pravenec M., Kurtz T.W. (2005). A new transgenic rat model of hepatic steatosis and the metabolic syndrome. Hypertension.

[266] Malínská H., Oliyarnyk O., Hubová M., Zídek V., Landa V., Simáková M., Mlejnek P., Kazdová L., Kurtz T.W., Pravenec M. (2010). Increased liver oxidative stress and altered PUFA metabolism precede development of non-alcoholic steatohepatitis in SREBP-1a transgenic spontaneously hypertensive rats with genetic predisposition to hepatic steatosis. Mol. Cell. Biochem..

[267] Takiguchi S., Takata Y., Funakoshi A., Miyasaka K., Kataoka K., Fujimura Y., Goto T., Kono A. (1997). Disrupted cholecystokinin type-A receptor (CCKAR) gene in OLETF rats. Gene.

[268] Song Y.S., Fang C.H., So B.I., Park J.Y., Lee Y., Shin J.H., Jun D.W., Kim H., Kim K.S. (2013). Time course of the development of nonalcoholic Fatty liver disease in the Otsuka long-evans Tokushima Fatty rat. Gastroenterol. Res. Pract.

[269] Uno M., Kurita S., Misu H., Ando H., Ota T., Matsuzawa-Nagata N., Kita Y., Nabemoto S., Akahori H., Zen Y. (2008). Tranilast, an antifibrogenic agent, ameliorates a dietary rat model of nonalcoholic steatohepatitis. Hepatology.

[270] Ota T., Takamura T., Kurita S., Matsuzawa N., Kita Y., Uno M., Akahori H., Misu H., Sakurai M., Zen Y. (2007). Insulin resistance accelerates a dietary rat model of nonalcoholic steatohepatitis. Gastroenterology.

[271] Kovar J., Tonar Z., Heczkova M., Poledne R. (2009). Prague hereditary hypercholesterolemic (PHHC) rat—A model of polygenic hypercholesterolemia. Physiol. Res..

[272] Befekadu G., Kovar J., Poledne R. (1992). High sensitivity of PHHC rat to dietary cholesterol. Physiol. Res..

[273] Yamashita T., Murakami T., Iida M., Kuwajima M., Shima K. (1997). Leptin receptor of Zucker fatty rat performs reduced signal transduction. Diabetes.

[274] Oana F., Takeda H., Hayakawa K., Matsuzawa A., Akahane S., Isaji M., Akahane M. (2005). Physiological difference between obese (fa/fa) Zucker rats and lean Zucker rats concerning adiponectin. Metabolism.

[275] Yang S.Q., Lin H.Z., Lane M.D., Clemens M., Diehl A.M. (1997). Obesity increases sensitivity to endotoxin liver injury: Implications for the pathogenesis of steatohepatitis. Proc. Natl. Acad. Sci. USA.

[276] Carmiel-Haggai M., Cederbaum A.I., Nieto N. (2005). A high-fat diet leads to the progression of non-alcoholic fatty liver disease in obese rats. FASEB J..

[277] Tan T., Song Z., Wang R., Jiang S., Liang Z., Wang Q., Hu X., Li N., Xing Y. (2021). Modelling Porcine NAFLD by Deletion of Leptin and defining the role of AMPK in hepatic fibrosis. bioRxiv.

[278] Wang H., Wu Y., Tang W. (2022). Methionine cycle in nonalcoholic fatty liver disease and its potential applications. Biochem. Pharmacol..

[279] Cong W.N., Tao R.Y., Tian J.Y., Liu G.T., Ye F. (2008). The establishment of a novel non-alcoholic steatohepatitis model accompanied with obesity and insulin resistance in mice. Life Sci..

[280] Kucsera D., Toth V.E., Gergo D., Voros I., Onodi Z., Gorbe A., Ferdinandy P., Varga Z.V. (2021). Characterization of the CDAA Diet-Induced Non-alcoholic Steatohepatitis Model: Sex-Specific Differences in Inflammation, Fibrosis, and Cholesterol Metabolism in Middle-Aged Mice. Front. Physiol..

[281] Chen H., Zhao W., Yan X., Huang T., Yang A. (2022). Overexpression of Hepcidin Alleviates Steatohepatitis and Fibrosis in a Diet-induced Nonalcoholic Steatohepatitis. J. Clin. Transl. Hepatol..

[282] Arman T., Lynch K.D., Montonye M.L., Goedken M., Clarke J.D. (2019). Sub-Chronic Microcystin-LR Liver Toxicity in Preexisting Diet-Induced Nonalcoholic Steatohepatitis in Rats. Toxins.

[283] Canet M.J., Hardwick R.N., Lake A.D., Dzierlenga A.L., Clarke J.D., Cherrington N.J. (2014). Modeling human nonalcoholic steatohepatitis-associated changes in drug transporter expression using experimental rodent models. Drug Metab. Dispos..

[284] Xin H.G., Zhang B.B., Wu Z.Q., Hang X.F., Xu W.S., Ni W., Zhang R.Q., Miao X.H. (2014). Treatment with baicalein attenuates methionine-choline deficient diet-induced non-alcoholic steatohepatitis in rats. Eur. J Pharmacol..

[285] Kirsch R., Clarkson V., Shephard E.G., Marais D.A., Jaffer M.A., Woodburne V.E., Kirsch R.E., Hall Pde L. (2003). Rodent nutritional model of non-alcoholic steatohepatitis: Species, strain and sex difference studies. J. Gastroenterol. Hepatol..

[286] Shimozato N., Namisaki T., Kaji K., Kitade M., Okura Y., Sato S., Moriya K., Seki K., Kawaratani H., Takaya H. (2019). Combined effect of a farnesoid X receptor agonist and dipeptidyl peptidase-4 inhibitor on hepatic fibrosis. Hepatol. Res..

[287] Hensley K., Kotake Y., Sang H., Pye Q.N., Wallis G.L., Kolker L.M., Tabatabaie T., Stewart C.A., Konishi Y., Nakae D. (2000). Dietary choline restriction causes complex I dysfunction and increased H_2_O_2_ generation in liver mitochondria. Carcinogenesis.

[288] Tamaki Y., Nakade Y., Yamauchi T., Makino Y., Yokohama S., Okada M., Aso K., Kanamori H., Ohashi T., Sato K. (2013). Angiotensin II type 1 receptor antagonist prevents hepatic carcinoma in rats with nonalcoholic steatohepatitis. J. Gastroenterol..

[289] Kitade M., Yoshiji H., Kojima H., Ikenaka Y., Noguchi R., Kaji K., Yoshii J., Yanase K., Namisaki T., Asada K. (2006). Leptin-mediated neovascularization is a prerequisite for progression of nonalcoholic steatohepatitis in rats. Hepatology.

[290] George J., Pera N., Phung N., Leclercq I., Yun Hou J., Farrell G. (2003). Lipid peroxidation, stellate cell activation and hepatic fibrogenesis in a rat model of chronic steatohepatitis. J. Hepatol..

[291] Cohen S.M., McQueen C.A. (2010). 14.11—The Role of Cell Proliferation in the Etiology of Neoplasia. Comprehensive Toxicology.

[292] Fu H., Tang B., Lang J., Du Y., Cao B., Jin L., Fang M., Hu Z., Cheng C., Liu X. (2020). High-Fat Diet Promotes Macrophage-Mediated Hepatic Inflammation and Aggravates Diethylnitrosamine-Induced Hepatocarcinogenesis in Mice. Front. Nutr..

[293] Arboatti A.S., Lambertucci F., Sedlmeier M.G., Pisani G., Monti J., Alvarez M.L., Frances D.E.A., Ronco M.T., Carnovale C.E. (2019). Diethylnitrosamine enhances hepatic tumorigenic pathways in mice fed with high fat diet (Hfd). Chem. Biol. Interact..

[294] Zhang C., Yang M. (2021). The Emerging Factors and Treatment Options for NAFLD-Related Hepatocellular Carcinoma. Cancers.

[295] Scholten D., Trebicka J., Liedtke C., Weiskirchen R. (2015). The carbon tetrachloride model in mice. Lab. Anim..

[296] Jacob M., Rani S.S., Shankar R., Raj A., Sujith S. (2022). An experiment-based approach for selecting optimal dosage of carbon tetrachloride for research studies on fatty liver disease. Pharma Innov. J..

[297] Somm E., Montandon S.A., Loizides-Mangold U., Gaia N., Lazarevic V., De Vito C., Perroud E., Bochaton-Piallat M.L., Dibner C., Schrenzel J. (2021). The GLP-1R agonist liraglutide limits hepatic lipotoxicity and inflammatory response in mice fed a methionine-choline deficient diet. Transl. Res..

[298] Miao H., Ouyang H., Guo Q., Wei M., Lu B., Kai G., Ji L. (2022). Chlorogenic acid alleviated liver fibrosis in methionine and choline deficient diet-induced nonalcoholic steatohepatitis in mice and its mechanism. J. Nutr. Biochem..

[299] Liao S., An K., Liu Z., He H., An Z., Su Q., Li S. (2022). Genetic variants associated with metabolic dysfunction-associated fatty liver disease in western China. J. Clin. Lab. Anal..

[300] Longo M., Meroni M., Paolini E., Erconi V., Carli F., Fortunato F., Ronchi D., Piciotti R., Sabatini S., Macchi C. (2022). TM6SF2/PNPLA3/MBOAT7 Loss-of-Function Genetic Variants Impact on NAFLD Development and Progression Both in Patients and in In Vitro Models. Cell. Mol. Gastroenterol. Hepatol..

[301] Linden D., Ahnmark A., Pingitore P., Ciociola E., Ahlstedt I., Andreasson A.C., Sasidharan K., Madeyski-Bengtson K., Zurek M., Mancina R.M. (2019). Pnpla3 silencing with antisense oligonucleotides ameliorates nonalcoholic steatohepatitis and fibrosis in Pnpla3 I148M knock-in mice. Mol. Metab..

[302] Xu X., Poulsen K.L., Wu L., Liu S., Miyata T., Song Q., Wei Q., Zhao C., Lin C., Yang J. (2022). Targeted therapeutics and novel signaling pathways in non-alcohol-associated fatty liver/steatohepatitis (NAFL/NASH). Signal Transduct. Target. Ther..

[303] Cherubini A., Casirati E., Tomasi M., Valenti L. (2021). PNPLA3 as a therapeutic target for fatty liver disease: The evidence to date. Expert. Opin. Ther. Targets.

[304] Bruschi F.V., Tardelli M., Claudel T., Trauner M. (2017). PNPLA3 expression and its impact on the liver: Current perspectives. Hepatic Med..

[305] Smagris E., BasuRay S., Li J., Huang Y., Lai K.M., Gromada J., Cohen J.C., Hobbs H.H. (2015). Pnpla3I148M knockin mice accumulate PNPLA3 on lipid droplets and develop hepatic steatosis. Hepatology.

[306] Nakagawa H., Umemura A., Taniguchi K., Font-Burgada J., Dhar D., Ogata H., Zhong Z., Valasek M.A., Seki E., Hidalgo J. (2014). ER stress cooperates with hypernutrition to trigger TNF-dependent spontaneous HCC development. Cancer Cell.

[307] Kim J.Y., He F., Karin M. (2021). From Liver Fat to Cancer: Perils of the Western Diet. Cancers.

[308] Green C.D., Weigel C., Brown R.D.R., Bedossa P., Dozmorov M., Sanyal A.J., Spiegel S. (2022). A new preclinical model of western diet-induced progression of non-alcoholic steatohepatitis to hepatocellular carcinoma. FASEB J..

[309] Sabir U., Irfan H.M., Alamgeer, Ullah A., Althobaiti Y.S., Asim M.H. (2022). Reduction of Hepatic Steatosis, Oxidative Stress, Inflammation, Ballooning and Insulin Resistance After Therapy with Safranal in NAFLD Animal Model: A New Approach. J. Inflamm. Res..

[310] Huby T., Gautier E.L. (2022). Immune cell-mediated features of non-alcoholic steatohepatitis. Nat. Rev. Immunol..

[311] Lee Y.A., Friedman S.L. (2022). Inflammatory and fibrotic mechanisms in NAFLD-Implications for new treatment strategies. J. Intern. Med..

[312] Gehrke N., Biedenbach J., Huber Y., Straub B.K., Galle P.R., Simon P., Schattenberg J.M. (2019). Voluntary exercise in mice fed an obesogenic diet alters the hepatic immune phenotype and improves metabolic parameters—An animal model of life style intervention in NAFLD. Sci. Rep..

[313] Aarts S., Reiche M., den Toom M., Gijbels M., Beckers L., Gerdes N., Lutgens E. (2019). Depletion of CD40 on CD11c(+) cells worsens the metabolic syndrome and ameliorates hepatic inflammation during NASH. Sci. Rep..

[314] Mendez-Sanchez N., Cordova-Gallardo J., Barranco-Fragoso B., Eslam M. (2021). Hepatic Dendritic Cells in the Development and Progression of Metabolic Steatohepatitis. Front. Immunol..

[315] Heier E.C., Meier A., Julich-Haertel H., Djudjaj S., Rau M., Tschernig T., Geier A., Boor P., Lammert F., Lukacs-Kornek V. (2017). Murine CD103(+) dendritic cells protect against steatosis progression towards steatohepatitis. J. Hepatol..

[316] Bhattacharjee J., Kumar J.M., Arindkar S., Das B., Pramod U., Juyal R.C., Majumdar S.S., Nagarajan P. (2014). Role of immunodeficient animal models in the development of fructose induced NAFLD. J. Nutr. Biochem..

[317] Her Z., Tan J.H.L., Lim Y.S., Tan S.Y., Chan X.Y., Tan W.W.S., Liu M., Yong K.S.M., Lai F., Ceccarello E. (2020). CD4(+) T Cells Mediate the Development of Liver Fibrosis in High Fat Diet-Induced NAFLD in Humanized Mice. Front. Immunol..

[318] Gheorghe C.E., Ritz N.L., Martin J.A., Wardill H.R., Cryan J.F., Clarke G. (2021). Investigating causality with fecal microbiota transplantation in rodents: Applications, recommendations and pitfalls. Gut Microbes.

[319] Romualdo G.R., Valente L.C., Sprocatti A.C., Bacil G.P., de Souza I.P., Rodrigues J., Rodrigues M.A.M., Vinken M., Cogliati B., Barbisan L.F. (2022). Western diet–induced mouse model of non-alcoholic fatty liver disease associated with metabolic outcomes: Features of gut microbiome-liver-adipose tissue axis. Nutrition.

[320] Qv L., Yang Z., Yao M., Mao S., Li Y., Zhang J., Li L. (2020). Methods for establishment and maintenance of germ-free rat models. Front. Microbiol..

[321] Uzbay T. (2019). Germ-free animal experiments in the gut microbiota studies. Curr. Opin. Pharmacol..

[322] Eberl C., Ring D., Münch P.C., Beutler M., Basic M., Slack E.C., Schwarzer M., Srutkova D., Lange A., Frick J.S. (2020). Reproducible colonization of germ-free mice with the oligo-mouse-microbiota in different animal facilities. Front. Microbiol..

[323] Rao Y., Kuang Z., Li C., Guo S., Xu Y., Zhao D., Hu Y., Song B., Jiang Z., Ge Z. (2021). Gut Akkermansia muciniphila ameliorates metabolic dysfunction-associated fatty liver disease by regulating the metabolism of L-aspartate via gut-liver axis. Gut Microbes.

[324] Matsui M., Fukunishi S., Nakano T., Ueno T., Higuchi K., Asai A. (2021). Ileal Bile Acid Transporter Inhibitor Improves Hepatic Steatosis by Ameliorating Gut Microbiota Dysbiosis in NAFLD Model Mice. mBio.

[325] Moreira G.V., Azevedo F.F., Ribeiro L.M., Santos A., Guadagnini D., Gama P., Liberti E.A., Saad M.J.A., Carvalho C.R.O. (2018). Liraglutide modulates gut microbiota and reduces NAFLD in obese mice. J. Nutr. Biochem..

[326] Kong C.-Y., Li Z.-M., Chen H.-L., Mao Y.-Q., Han B., Guo J.-J., Wang L.-S. (2022). An Energy-Restricted Diet Including Yogurt, Fruit, and Vegetables Alleviates High-Fat Diet–Induced Metabolic Syndrome in Mice by Modulating the Gut Microbiota. J. Nutr..

[327] Hou D., Tang J., Huan M., Liu F., Zhou S., Shen Q. (2022). Alteration of fecal microbiome and metabolome by mung bean coat improves diet-induced non-alcoholic fatty liver disease in mice. Food Sci. Hum. Wellness.

[328] Zhai Y., Zhou W., Yan X., Qiao Y., Guan L., Zhang Z., Liu H., Jiang J., Liu J., Peng L. (2022). Astragaloside IV ameliorates diet-induced hepatic steatosis in obese mice by inhibiting intestinal FXR via intestinal flora remodeling. Phytomedicine.

[329] Sakurai T., Hayasaka T., Sekiguchi H., Satoh H., Chen Z., Chiba H., Hui S.P. (2019). Dietary salmon milt extracts attenuate hepatosteatosis and liver dysfunction in diet-induced fatty liver model. J. Sci. Food Agric..

[330] Li X., Zhao W., Xiao M., Yu L., Chen Q., Hu X., Zhao Y., Xiong L., Chen X., Wang X. (2022). Penthorum chinense Pursh. extract attenuates non-alcholic fatty liver disease by regulating gut microbiota and bile acid metabolism in mice. J. Ethnopharmacol..

[331] Meng W., Zhao Z., Chen L., Lin S., Zhang Y., He J., Ouyang K., Wang W. (2022). Total Flavonoids from Chimonanthus nitens Oliv. Leaves Ameliorate HFD-Induced NAFLD by Regulating the Gut–Liver Axis in Mice. Foods.

[332] Luo D., Yang L., Pang H., Zhao Y., Li K., Rong X., Guo J. (2022). Tianhuang formula reduces the oxidative stress response of NAFLD by regulating the gut microbiome in mice. Front Microbiol.

[333] Kawano Y., Edwards M., Huang Y., Bilate A.M., Araujo L.P., Tanoue T., Atarashi K., Ladinsky M.S., Reiner S.L., Wang H.H. (2022). Microbiota imbalance induced by dietary sugar disrupts immune-mediated protection from metabolic syndrome. Cell.

[334] Pingitore P., Romeo S. (2019). The role of PNPLA3 in health and disease. Biochim. Biophys. Acta Mol. Cell Biol. Lipids.

[335] Yu W., Lei Q., Yang L., Qin G., Liu S., Wang D., Ping Y., Zhang Y. (2021). Contradictory roles of lipid metabolism in immune response within the tumor microenvironment. J. Hematol. Oncol..

[336] Aron-Wisnewsky J., Vigliotti C., Witjes J., Le P., Holleboom A.G., Verheij J., Nieuwdorp M., Clément K. (2020). Gut microbiota and human NAFLD: Disentangling microbial signatures from metabolic disorders. Nat. Rev. Gastroenterol. Hepatol..

[337] Gupta H., Min B.-H., Ganesan R., Gebru Y.A., Sharma S.P., Park E., Won S.-M., Jeong J.-J., Lee S.-B., Cha M.-G. (2022). Gut Microbiome in Non-Alcoholic Fatty Liver Disease: From Mechanisms to Therapeutic Role. Biomedicines.

[338] Sanyal A.J. (2019). Past, present and future perspectives in nonalcoholic fatty liver disease. Nat. Rev. Gastroenterol. Hepatol..

[339] Itagaki H., Shimizu K., Morikawa S., Ogawa K., Ezaki T. (2013). Morphological and functional characterization of non-alcoholic fatty liver disease induced by a methionine-choline-deficient diet in C57BL/6 mice. Int. J. Clin. Exp. Pathol..

[340] Rinella M.E., Green R.M. (2004). The methionine-choline deficient dietary model of steatohepatitis does not exhibit insulin resistance. J. Hepatol..

[341] Pellizzon M.A., Ricci M.R. (2020). Choice of Laboratory Rodent Diet May Confound Data Interpretation and Reproducibility. Curr. Dev. Nutr..

[342] Pellizzon M.A., Ricci M.R. (2018). The common use of improper control diets in diet-induced metabolic disease research confounds data interpretation: The fiber factor. Nutr. Metab..

[343] Warden C.H., Fisler J.S. (2008). Comparisons of diets used in animal models of high-fat feeding. Cell Metab..

[344] Pellizzon M. (2016). Choice of laboratory animal diet influences intestinal health. Lab. Anim..

[345] Abu-Shanab A., Quigley E.M. (2010). The role of the gut microbiota in nonalcoholic fatty liver disease. Nat. Rev. Gastroenterol. Hepatol..

[346] Lian C.Y., Zhai Z.Z., Li Z.F., Wang L. (2020). High fat diet-triggered non-alcoholic fatty liver disease: A review of proposed mechanisms. Chem. Biol. Interact..

[347] Buettner R., Scholmerich J., Bollheimer L.C. (2007). High-fat diets: Modeling the metabolic disorders of human obesity in rodents. Obesity.

[348] Buzzetti E., Pinzani M., Tsochatzis E.A. (2016). The multiple-hit pathogenesis of non-alcoholic fatty liver disease (NAFLD). Metabolism.

[349] Cronin P., Joyce S.A., O’Toole P.W., O’Connor E.M. (2021). Dietary Fibre Modulates the Gut Microbiota. Nutrients.

[350] Seeley R.J., MacDougald O.A. (2021). Mice as experimental models for human physiology: When several degrees in housing temperature matter. Nat. Metab..

[351] Tian X.Y., Ganeshan K., Hong C., Nguyen K.D., Qiu Y., Kim J., Tangirala R.K., Tontonoz P., Chawla A. (2016). Thermoneutral Housing Accelerates Metabolic Inflammation to Potentiate Atherosclerosis but Not Insulin Resistance. Cell Metab..

[352] Cannon B., Nedergaard J. (2011). Nonshivering thermogenesis and its adequate measurement in metabolic studies. J. Exp. Biol..

[353] Giles D.A., Moreno-Fernandez M.E., Stankiewicz T.E., Graspeuntner S., Cappelletti M., Wu D., Mukherjee R., Chan C.C., Lawson M.J., Klarquist J. (2017). Thermoneutral housing exacerbates nonalcoholic fatty liver disease in mice and allows for sex-independent disease modeling. Nat. Med..

[354] Worthmann A., John C., Ruhlemann M.C., Baguhl M., Heinsen F.A., Schaltenberg N., Heine M., Schlein C., Evangelakos I., Mineo C. (2017). Cold-induced conversion of cholesterol to bile acids in mice shapes the gut microbiome and promotes adaptive thermogenesis. Nat. Med..

[355] Cui X., Nguyen N.L., Zarebidaki E., Cao Q., Li F., Zha L., Bartness T., Shi H., Xue B. (2016). Thermoneutrality decreases thermogenic program and promotes adiposity in high-fat diet-fed mice. Physiol. Rep..

[356] Montandon S.A., Somm E., Loizides-Mangold U., de Vito C., Dibner C., Jornayvaz F.R. (2019). Multi-technique comparison of atherogenic and MCD NASH models highlights changes in sphingolipid metabolism. Sci. Rep..

[357] Long M.T., Noureddin M., Lim J.K. (2022). AGA Clinical Practice Update: Diagnosis and management of nonalcoholic fatty liver disease in lean individuals: Expert review. Gastroenterology.

[358] Tang A., Ng C.H., Phang P.H., Chan K.E., Chin Y.H., Fu C.E., Zeng R.W., Xiao J., Tan D.J.H., Quek J. (2022). Comparative Burden of Metabolic Dysfunction in Lean NAFLD vs. Non-Lean NAFLD-A Systematic Review and Meta-Analysis. Clin. Gastroenterol. Hepatol..

[359] Xu R., Pan J., Zhou W., Ji G., Dang Y. (2022). Recent advances in lean NAFLD. Biomed. Pharmacother..

[360] Dufour J.-F., Anstee Q.M., Bugianesi E., Harrison S., Loomba R., Paradis V., Tilg H., Wong V.W.-S., Zelber-sagi S. (2022). Current therapies and new developments in NASH. Gut.

[361] Guirguis E., Grace Y., Bolson A., DellaVecchia M.J., Ruble M. (2021). Emerging therapies for the treatment of nonalcoholic steatohepatitis: A systematic review. Pharmacother. J. Hum. Pharmacol. Drug Ther..

[362] Sharma M., Premkumar M., Kulkarni A.V., Kumar P., Reddy D.N., Rao N.P. (2021). Drugs for Non-alcoholic Steatohepatitis (NASH): Quest for the Holy Grail. J. Clin. Transl. Hepatol..

[363] Cataldo I., Sarcognato S., Sacchi D., Cacciatore M., Baciorri F., Mangia A., Cazzagon N., Guido M. (2021). Pathology of non-alcoholic fatty liver disease. Pathologica.

[364] Lin S.Z., Chen Y.W., Fan J.G. (2020). Non-alcoholic fatty liver disease to metabolic dysfunction-associated fatty liver disease: Conceptual changes for clinicians, researchers and patients. J. Dig. Dis..

[365] Hu C., Jia W. (2021). Multi-omics profiling: The way toward precision medicine in metabolic diseases. J. Mol. Cell Biol..

[366] Sveinbjornsson G., Ulfarsson M.O., Thorolfsdottir R.B., Jonsson B.A., Einarsson E., Gunnlaugsson G., Rognvaldsson S., Arnar D.O., Baldvinsson M., Bjarnason R.G. (2022). Multiomics study of nonalcoholic fatty liver disease. Nat. Genet..

[367] Ghallab A., Myllys M., Friebel A., Duda J., Edlund K., Halilbasic E., Vucur M., Hobloss Z., Brackhagen L., Begher-Tibbe B. (2021). Spatio-Temporal Multiscale Analysis of Western Diet-Fed Mice Reveals a Translationally Relevant Sequence of Events during NAFLD Progression. Cells.

[368] Hundertmark J., Tacke F. (2020). How effective are nonalcoholic fatty liver disease models for drug discovery?. Expert Opin. Drug Discov..

[369] Kalogirou M.-S., Patoulias D., Haidich A.-B., Akriviadis E., Sinakos E. (2021). Liraglutide in patients with non-alcoholic fatty liver disease: A systematic review and meta-analysis of randomized controlled trials. Clin. Res. Hepatol. Gastroenterol..

[370] Van Wagner L.B., Koppe S.W., Brunt E.M., Gottstein J., Gardikiotes K., Green R.M., Rinella M.E. (2016). Pentoxifylline for the treatment of non-alcoholic steatohepatitis: A randomized controlled trial. Ann. Hepatol..

[371] Seko Y., Yamaguchi K., Yano K., Takahashi Y., Takeuchi K., Kataoka S., Moriguchi M., Itoh Y. (2022). The additive effect of genetic and metabolic factors in the pathogenesis of nonalcoholic fatty liver disease. Sci. Rep..

[372] De Vincentis A., Tavaglione F., Jamialahmadi O., Picardi A., Antonelli Incalzi R., Valenti L., Romeo S., Vespasiani-Gentilucci U. (2022). A Polygenic Risk Score to Refine Risk Stratification and Prediction for Severe Liver Disease by Clinical Fibrosis Scores. Clin. Gastroenterol. Hepatol..

